# A versatile nanobody platform for live and super-resolution imaging of synaptic vesicle dynamics and plasticity in rodent and human neurons

**DOI:** 10.1186/s12951-026-04489-w

**Published:** 2026-05-14

**Authors:** Rashi Goel, Kristina Jevdokimenko, Ronja Rehm, Jannik Hentze, Paola Agüi-Gonzalez, Momchil Ninov, Erik Maier, Yin Wu, Felix Lange, Agata Witkowska, Svenja Bolz, Francesca Pennacchietti, Martina Damenti, Natalie Kaempf, Vladimir Khayenko, Carles Calatayud, Viveka Nand Malviya, Natali L. Chanaday, Emma Scaletti Hutchinson, Hao Liu, Kirsten Weyand, Valentin Schwarze, Daniela Ivanova, Tristan P. Wallis, Christopher Small, Hans M. Maric, Merja Joensuu, Michael A. Cousin, Frédéric A. Meunier, Patrik Verstreken, Ilaria Testa, Ege T. Kavalali, Volker Haucke, Stefan Jakobs, Henning Urlaub, Nils Brose, Benjamin H. Cooper, Pål Stenmark, Felipe Opazo, Reinhard Jahn, Silvio O. Rizzoli, Eugenio F. Fornasiero

**Affiliations:** 1 Laboratory of Neurobiology, Max Planck Institute for Multidisciplinary Sciences, 37077 Göttingen, Germany; 2 Institute of Neuro- and Sensory Physiology, University Medical Center Göttingen, 37073 Göttingen, Germany; 3 Center for Biostructural Imaging of Neurodegeneration (BIN), University Medical Center Göttingen, 37075 Göttingen, Germany; 4 Bioanalytical Mass Spectrometry Research Group, Max Planck Institute for Multidisciplinary Sciences, 37077 Göttingen, Germany; 5 Department of NanoBiophotonics, Max Planck Institute for Multidisciplinary Sciences, 37077 Göttingen, Germany; 6 Clinic of Neurology, University Medical Center Göttingen, 37075 Göttingen, Germany; 7 Leibniz-Forschungsinstitut für Molekulare Pharmakologie (FMP), 13125 Berlin, Germany; 8 NeuroCure Cluster of Excellence, Charité Universitätsmedizin Berlin, 10117 Berlin, Germany; 9 Department of Applied Physics and SciLifeLab, KTH Royal Institute of Technology, Stockholm, 17165 Sweden; 10 8 VIB-KU Leuven Center for Neuroscience, Leuven, 3000 Belgium; 11 Department of Neurosciences, Leuven Brain Institute, Belgium and KU Leuven, Leuven, 3000 Belgium; 12 Rudolf Virchow Center, Center for Integrative and Translational Bioimaging, University of Wuerzburg, 97080 Wuerzburg, Germany; 13 Department of Physiology, Perelman School of Medicine, University of Pennsylvania, Philadelphia, PA 19104 USA; 14 Department of Biochemistry and Biophysics, Stockholm University, Stockholm, SE-106 91 Sweden; 15 Department of Molecular Neurobiology, Max Planck Institute for Multidisciplinary Sciences, 37075 Göttingen, Germany; 16 Centre for Discovery Brain Sciences, University of Edinburgh, Hugh Robson Building, George Square, Edinburgh, Scotland UK; 17 Simons Initiative for the Developing Brain, University of Edinburgh, Hugh Robson Building, Edinburgh, EH8 9XD Scotland UK; 18 Muir Maxwell Epilepsy Centre, University of Edinburgh, Hugh Robson Building, Edinburgh, EH8 9XD Scotland UK; 19 Clem Jones Centre for Ageing Dementia Research, Queensland Brain Institute, The University of Queensland, Brisbane, QLD 4072 Australia; 20 Australian Institute for Bioengineering and Nanotechnology, The University of Queensland, Brisbane, QLD 4072 Australia; 21 School of Biomedical Sciences, The University of Queensland, Brisbane, QLD 4072 Australia; 22 Department of Pharmacology, School of Medicine, Vanderbilt University, Nashville, TN 37240-7933 USA; 23 Vanderbilt Brain Institute, Vanderbilt University, Nashville, TN 37240-7933 USA; 24 Faculty of Biology, Freie Universität Berlin, 14195 Chemistry, Pharmacy, Berlin, Germany; 25 Department of NanoBiophotonics, Max Planck Institute for Multidisciplinary Sciences, 37077 Göttingen, Germany; 26 Clinic of Neurology, University Medical Center Göttingen, 37075 Göttingen, Germany; 27 Fraunhofer Institute for Translational Medicine and Pharmacology ITMP,, Translational Neuroinflammation and Automated Microscopy, 37075 Göttingen, Germany; 28 Bioanalytics, Department of Clinical Chemistry, University Medical Center Göttingen, 37075 Göttingen, Germany; 29 Cluster of Excellence ″Multiscale Bioimaging: from Molecular Machines to Networks of Excitable Cells″ (MBExC), University of Göttingen, 37075 Göttingen, Germany; 30 NanoTag Biotechnologies GmbH, 37079 Göttingen, Germany; 31 Department of Life Sciences, University of Trieste, Trieste, 34127 Italy

**Keywords:** Nanotools, Synaptic vesicles, Synaptotagmin, Presynaptic biology, Nanobodies, SdAb, Biomedical engineering

## Abstract

Synaptic neurotransmission is a critical hallmark of brain activity and one of the first processes affected in neural diseases. Monitoring this process, particularly synaptic vesicle recycling, in living cells has been instrumental in revealing the mechanisms responsible for neurotransmitter release. However, currently available reporters suffer from limitations, such as large probe sizes or limited compatibility for human neurons, hampering the quantitative analysis of synaptic pathophysiology. Here, we describe the NbLumSyt1 toolkit, a panel of nanobody-based affinity probes that target the luminal domain of the synaptic vesicle protein Synaptotagmin 1 (Syt1). These new tools enable quantitative, noninvasive imaging and functional interrogation of Syt1 exo-endocytosis and trafficking in human neurons, with unprecedented precision, versatility and cost efficiency, in technologies ranging from fixed- and live-cell super-resolution imaging to electron microscopy and mass spectrometry. Overall, NbLumSyt1 nanobinders provide a valuable platform for studying synaptic physiology and pathophysiology, benefiting fundamental neuroscience and translational efforts to study and develop treatments for brain-related disorders.

**Graphical Abstract:**

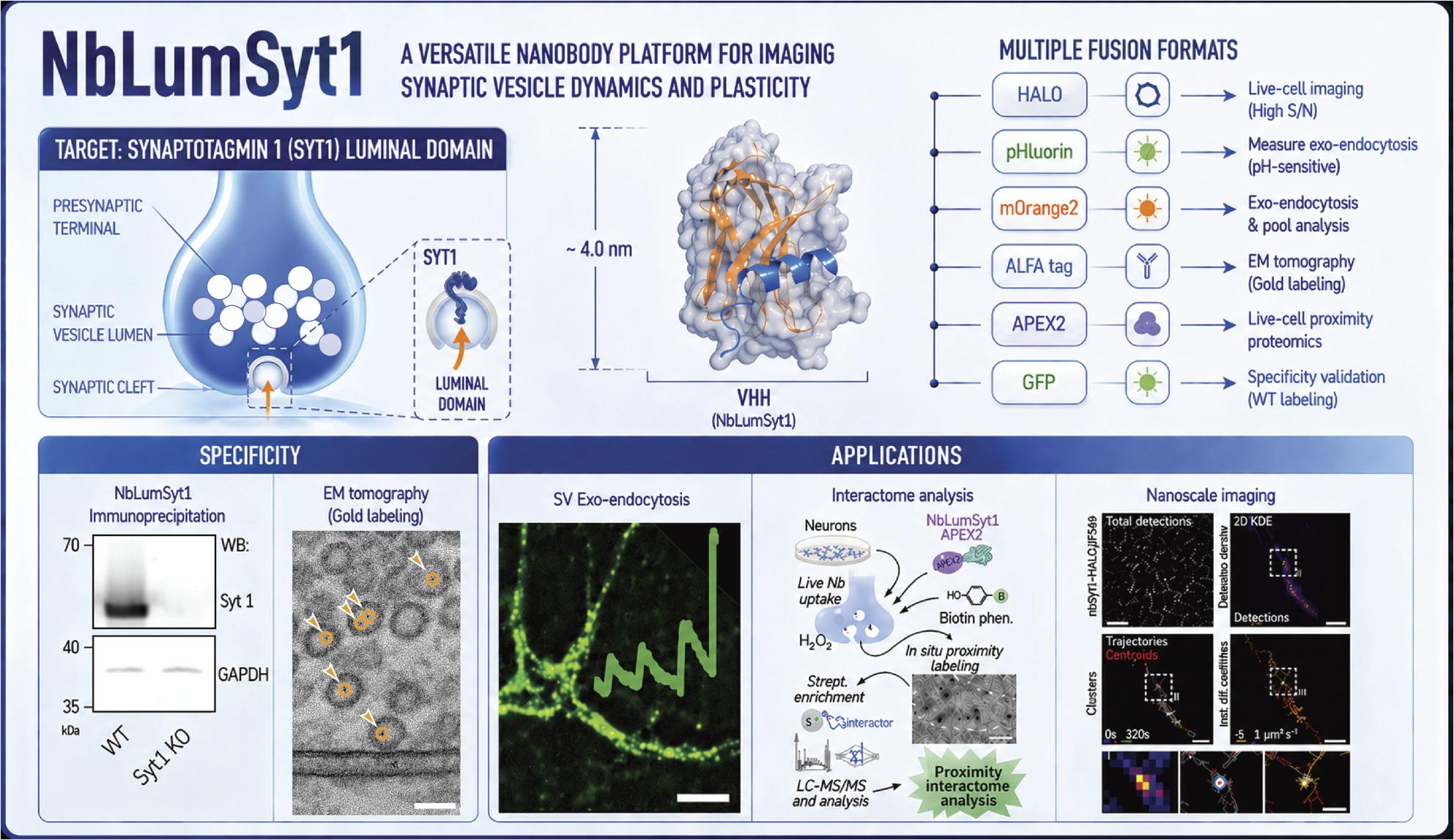

**Supplementary Information:**

The online version contains supplementary material available at 10.1186/s12951-026-04489-w.

## Introduction

Synaptic vesicle (SV) recycling, one of the most complex and well-regulated processes at neuronal presynapses, is responsible for the efficient and rapid release of neurotransmitters by exocytosis and the subsequent reuse of SVs [1]. It is essential for brain function, ensures the maintenance of neuronal homeostasis, and plays a leading role in cognitive processes such as learning and memory and in keeping neuronal circuits functionally intact [2]. Many neurodevelopmental and neurodegenerative diseases are linked to mutations in proteins essential for SV recycling [3, 4]. Rodent models have contributed significantly to our understanding of synaptic physiology, but growing evidence points to distinctive features of human neurons that are not fully captured in rodents [5], making direct investigation of human cells essential for studying synaptic pathologies linked to cognitive decline. Despite its importance, little is known about SV recycling in human neurons, partly because appropriate tools for analyzing this process are lacking.

Synaptotagmin 1 (Syt1) is a calcium-binding protein that plays a critical role in both exocytosis and endocytosis of SVs [6] and has been used as a key target for studying SV recycling [7]. Antibodies (IgGs) [8] are limited by their relatively large size (linkage error) and bivalency. For example, targeting multiple IgGs (diameter ~ 9–14 nm) to the lumen of SVs (~ 40 nm [8–11]) is challenging. In addition, this method can artificially cluster unfixed molecules in live assays [12].

Camelid single-domain antibodies, often also referred to as nanobodies, offer a promising alternative to conventional IgGs. Owing to their small size of ~ 15 kDa, nanobodies can reach epitopes that are sterically difficult to access with conventional antibodies [13]. Moreover, their excellent stability, ease of engineering, and low-cost production render nanobodies attractive probes for both basic research and clinical applications [14, 15].

In this work, we describe the generation and validation of a panel of nanobodies selective for the luminal domain of Syt1, which we optimized into a diverse array of probes collectively referred to as the NbLumSyt1 toolkit. These nanobodies allow the precise and minimally invasive study of SV recycling and neuronal function in both rodent and human neurons. Our NbLumSyt1-based probes display excellent performance in live-cell labeling and can be adapted to various experimental workflows, including single-molecule tracking, super-resolution imaging modalities and live-cell proteomic mapping, circumventing the classical limitations of conventional IgGs.

## Results

### Development of a nanobody targeting the luminal domain of Syt1

**Fig. 1:**
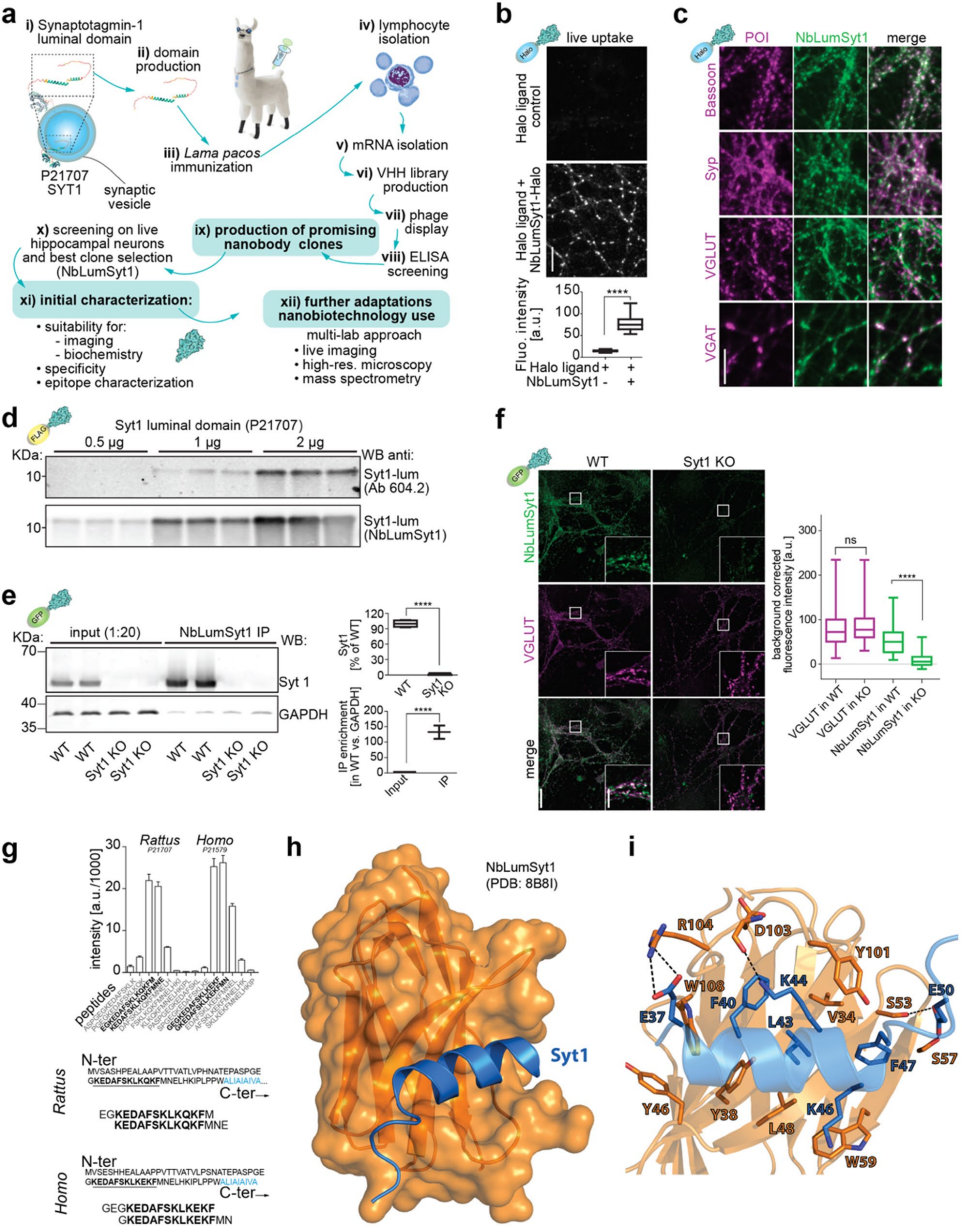
Development and characterization of a nanobody against the luminal domain of the calcium sensor Synaptotagmin **1. a**) Schematic representation of nanobody selection and characterization. **b**) Immunofluorescence images of live-labeled hippocampal neurons with NbLumSyt1 fused to the Halo-Tag. This tool can be used to label actively recycled synaptic vesicles and provides excellent signal-to-noise images. **c**) In live imaging and retrospective immunofluorescence, NbLumSyt1 colocalizes with the presynaptic scaffold protein Bassoon and labels synaptic boutons, including excitatory (VGLUT^+^) and inhibitory (VGAT^+^) boutons. **d**) Western blot analysis of purified Syt1. The nanobody can be used in WB applications and recognizes increasing concentrations of purified Syt1. **e**) Syt1 immunoprecipitation in WT or Syt1 KO mouse primary neurons using the nanobody, followed by western blot using an Syt1 antibody. Note that the smear-like effect in the IP for the WT samples is likely due to gel overloading. **f**) Immunofluorescence imaging following nanobody uptake in WT and Syt1 KO primary neurons and post-fixation staining of VGLUT. **g**) A peptide microarray binding assay with synthesized and immobilized rat and human Syt1 protein sequences identified the region of Syt1 that the nanobody binds to in both species. **h**) Crystal structure of the nanobody (orange) bound to Syt1 (blue). **i**) Specific amino acids involved in nanobody-Syt1 binding (PDB: 8B8I). Scale bars: 10 μm in **b**, **c**, and **f**-inset; 30 μm in **f**. The error bars indicate the means ± SEMs for panels e and g and the 5th to 95th percentiles for the boxplot in panel f; ns, not significant, **** *p* < 0.0001

A nanobody library was generated from peripheral blood mononuclear cells from alpacas immunized with a recombinant protein fragment corresponding to the luminal domain of rat Syt1 (Fig. 1a). Clones were screened for endocytic uptake in cultured hippocampal neurons (Supplementary Fig. 1a). Clone 1F12 displayed clear synaptic localization and was named NbLumSyt1. A version of NbLumSyt1 fused to a HALO-Tag [16] allowed live-cell imaging with high signal-to-noise ratios (Fig. 1a, b). The NbLumSyt1-HALO specifically labeled presynaptic boutons, colocalized with the presynaptic scaffold Bassoon and labeled both excitatory (VGLUT-positive) and inhibitory (VGAT-positive) synapses (Fig. 1c). In Western blot (WB) experiments, the sensitivity of the nanobody toward purified Syt1 was comparable to that of an established monoclonal antibody raised against the same antigen (Fig. 1d). Two orthogonal approaches demonstrated NbLumSyt1 specificity: (i) NbLumSyt1 reliably immunoprecipitates endogenous Syt1 from wild-type (WT), but not Syt1 KO [17], neurons (Fig. 1e), and (ii) NbLumSyt1 fused to a GFP tag fluorescently labels WT, but not Syt1 KO, neurons (Fig. 1f).

Epitope mapping of NbLumSyt1 with a peptide microarray [18] revealed a luminal region of Syt1 conserved between rodents and humans (Fig. 1g). Notably, immunoreactivity was lost after chemical fixation, possibly due to derivatization of conserved lysine residues in the epitope (Supplementary Fig. 1b, c). To further demonstrate the use of these nanotools, we validated the colocalization between different fusion versions of the NbLumSyt1 and the signal from an antibody targeting the cytoplasmatic domain of Syt1 (Supplementary Fig. 1d).

For additional characterization, we also solved the structure of NbLumSyt1 in complex with the identified Syt1 peptide via X-ray crystallography at a resolution of 2.75 Å (Fig. 1h, i; PDB: 8B8I). NbLumSyt1 displays a canonical IgG fold, where a disulfide bond between Cys23 and Cys96 connects two β-sheets. The Syt1 peptide forms a helical structure and binds to a shallow pocket on the surface of the nanobody. This area is primarily comprised of complementarity-determining regions (CDRs) 1, 2 and 3. The Syt1 peptide is positioned by a salt bridge between Glu37 and Arg104 of NbLumSyt1. Hydrogen bond interactions exist between Lys44 and Asp103 and between Glu50 and Ser53/Ser57 of NbLumSyt1. Residues Phe40, Leu43 and Phe47 from Syt1 form hydrophobic interactions with several hydrophobic amino acids in the nanobody, including Val34, Tyr46, Trp59, and Trp108. Analysis of the nanobody binding interface with protein interfaces, surfaces, and assemblies (PISA [19]) demonstrated a solvation-free energy gain of 10.3 kcal/mol, with an interface area of 772 Å^2^.

Overall, these experiments establish the NbLumSyt1 as a specific and well-validated nanobody against the luminal domain of Syt1, enabling selective labeling in live neurons, supported by complementary biochemical, imaging, and structural evidence.

### 3D ultrastructural analysis of synaptic vesicle recycling

**Fig. 2:**
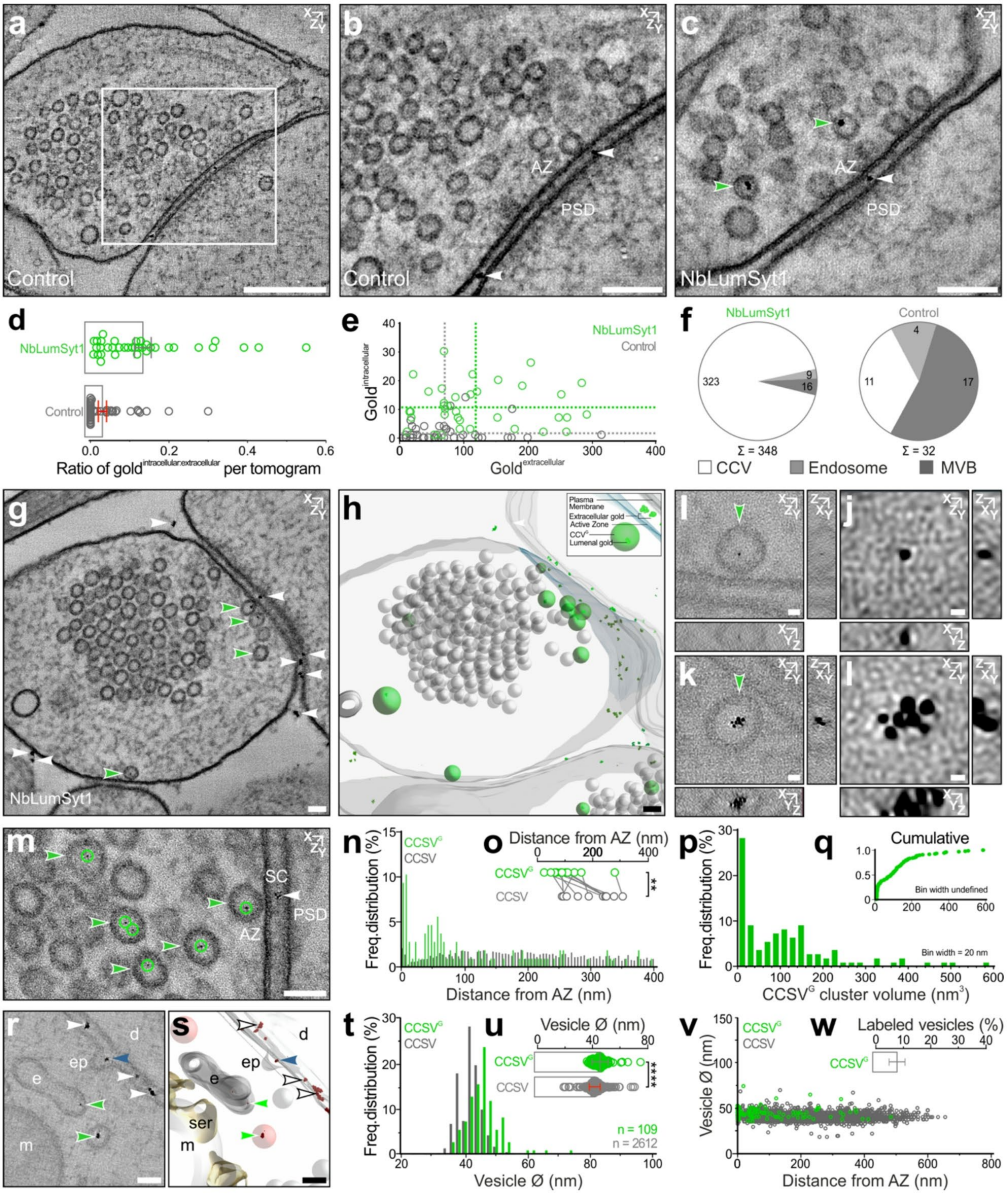
3D ultrastructural analysis of live-cell uptake of the NbLumSyt1-ALFA-tag: NbALFA-Gold complex. **a-c**) Electron tomographic subvolumes acquired from synapses at 36000x magnification (voxel size x, y,z = 1.2 nm; z-projection = 6 slices) from ALFA-Gold (a, b; Control) and NbLumSyt1-ALFA-tag: NbALFA-Gold complex (c; NbLumSyt1) treated conditions. The white box represents the presynaptic active zone enlarged in b. Note that the plane chosen in b differs from that in panel a. **d**) Ratio of intracellular (gold^intracellular^) to extracellular (gold^extracellular^) gold for each tomogram. **e**) Absolute numbers of gold clusters found intracellularly versus extracellularly for each tomogram. The dotted lines indicate the mean values for the control (gray; Gold^intracellular^ mean ± SEM = 1.95 ± 0.52; Gold^extracellular^, mean ± SEM = 70.60 ± 10.40) and NbLumSyt1-ALFA-tag: NbALFA-Gold complex (green; Gold^intracellular^ mean ± SEM = 11.00 ± 1.24; Gold^extracellular^, mean ± SEM = 118.10 ± 14.07) conditions. **f**) Presynaptic distribution of gold clusters within different organelles: clear core SVs (CCSVs, white), endosomes (light gray) and multivesicular bodies (MVBs, dark gray). **g-h**) Electron tomographic subvolume acquired from a synapse at 57000x magnification (voxel size x, y,z = 1 nm; z-projection = 20 slices) from the NbLumSyt1-ALFA-tag: NbALFA-Gold complex (**g**; NbLumSyt1)-treated condition and the corresponding 3D model (**h**). [Clear-core vesicles, CCSV (gray spheres); gold-labeled clear-core vesicles, CCSV^G^ (green spheres); gold clusters (green); active zone, AZ (blue); plasma membrane (gray); white and green arrowheads indicate extracellularly and intracellularly localized gold clusters, respectively]. (**i-l**) Orthogonal perspectives of a CCSV^G^ containing either single (**I**) or multiple (**k**) 3 nm gold particles (**j**, **l**, enlargements; voxel size x, y,z = 0.5 nm; z-projection = 3 slices). **m)** Tomographic subvolume revealing gold-labeled vesicles accumulated in close proximity to the active zone [CCSV^G^, green arrowheads; luminal gold particles, green circles; extracellular gold, white arrowheads]. **n**,** o**) Spatial distribution of gold-labeled synaptic vesicles (CCSV^G^, green) and unlabeled vesicles (CCSV, gray) within 400 nm from the AZ (5 nm bins) (**n**). Mean distance of CCSV and CCSV^G^ populations from the AZ quantified for each tomogram (**o**) (CCSV^G^, mean ± SEM = 106.50 ± 20.72 nm; CCSV, mean ± SEM = 199.30 ± 23.53 nm; paired Student’s t test, *p* = 0.0052). **p**,** q**) Volume of CCSV^G^ luminal gold clusters represented as frequency distribution (**p**; bin width = 20 nm^3^) and cumulative probability plots (**q**; bin width undefined). **r**,** s**) Tomographic subvolume (**r**; binned voxel size x, y,z = 1.2 nm; z-projection = 6 slices) and 3D model (**s**) in which multiple gold-labeled endocytic intermediates are captured (white arrowheads, extracellular gold; green arrowheads, endocytosed gold; blue arrowhead, gold localized to the developing lumen of an endocytic pit). **t**,** u**) Frequency distribution (**t**, bin width = 2 nm) and corresponding scatterplot (**u**) indicating the diameters of all quantified CCSV^G^ (green, *n* = 109 vesicles) and CCSV (gray, *n* = 2612 vesicles) diameters (CCSV^G^, mean ± SEM = 45.71 ± 0.56 nm; CCSV, mean ± SEM = 41.75 ± 0.08 nm; Kolmogorov‒Smirnov test, *p* < 0.0001). **v**) Spatial distributions of CCSV^G^ (green) and CCSV (gray) relative to the AZ and respective vesicle diameters (Ø). **w**) Percentage of labeled vesicles (CCSV^G^) quantified per analyzed tomogram. Abbreviations: Active zone, AZ; synaptic cleft, SC; postsynaptic density, PSD; dendrite, d; endocytic pit, ep; endosome, e; smooth endoplasmic reticulum, ser; mitochondria, m. Scale bars: 250 nm in **a**; 100 nm in **b** and **c**;50 nm in **g**, **h**, **m**, **r**, and **s**; 10 nm in **i** and **k**; 3 nm in **j** and **l**. Error bars indicate the means ± SEMs; * *p* < 0.05, ** *p* < 0.01, **** *p* < 0.0001. (**a-g**) 36000x magnification tomograms from two independent cultures for the control (*n* = 39 tomograms) and NbLumSyt1 (*n* = 36 tomograms) **(h-w)** 57000x magnification tomograms from one culture; NbLumSyt1 (*n* = 11 tomograms)

For electron microscopy applications, we generated a gold-labeled version of NbLumSyt1 by fusing it to ALFA-tag [20] and allowing complexation with NbALFA-Gold. Cultured hippocampal neurons were incubated with the labeled complex and then analyzed via EM tomography. The high spatial resolution of the internalized gold label revealed their localization in vesicles within presynaptic terminals (Fig. 2). Compared with controls treated with the same concentration of NbALFA-Gold, a significantly greater percentage of tomograms presented intracellular gold labeling with the NbLumSyt1-ALFA-tag: NbALFA-Gold complex (NbLumSyt1, 100%; control, 53,85%), along with an increased ratio of intracellular to extracellular gold labeling (Fig. 2a-e).

Absolute counts of the intracellular and extracellular gold particles further confirmed these results with significantly more internalized presynaptic gold particles detected in neurons treated with the NbLumSyt1-ALFA-tag: NbALFA-Gold complex compared to controls (NbLumSyt1, 348; control, 32). Intracellular gold particles within the presynapse predominantly labeled clear core SVs (CCSVs), but gold was also detected in endosomes and multivesicular bodies (Fig. 2f). Postsynaptic labeling, either at the plasma membrane or within the postsynaptic compartment, accounts for 12% of the signal in synapses treated with the NbLumSyt1-ALFA-tag: NbALFA-Gold complex, consistent with reports that Syt1 can also be detected postsynaptically [21], compared with 44% in synapses treated with the non-specific ALFA-gold control. The number of gold particles per cluster and the volume of individual gold clusters were comparable for intravesicular and extracellular labeling detected in NbLumSyt1 and control synapses, indicating that gold cluster dimensions per se do not provide a reliable quantitative assessment of Syt1 molecules per labeled vesicle. Accordingly, the number of luminal gold particles detected per vesicle should be interpreted as a relative indicator of vesicle recycling and labeling probability, rather than an absolute measure of Syt1 copy number.

At high magnification, individual SVs containing luminal gold particles were resolved, and tomographic reconstructions revealed their preferential accumulation near the active zone (Fig. 2g-m). These gold-labeled vesicles, hereafter referred to as CCSV^G^s, were selectively enriched closer to active zone (AZ) release sites compared to unlabeled SVs, consistent with their preferential recruitment during recycling (Fig. 2n-o). Additional tomograms provided further evidence for the specificity of gold labeling. Individual CCSV^G^s containing single or clustered gold particles were quantified, and unbiased volume estimates revealed a linear relationship between the number of luminal gold particles and the estimated gold cluster volume (Supplementary Fig. 2a-c).

Orthogonal views obtained from unbinned tomographic subvolumes demonstrate that ET can accurately visualize NbLumSyt1-ALFA-tag: NbALFA-Gold at single gold particle resolution (Ø = 3 nm; Supplementary Fig. 2d-f).

Quantification of the spatial distribution of presynaptic vesicles revealed that CCSV^G^s were positioned closer to the AZ than unlabeled CCSVs (Fig. 2n-o), indicating that recycling vesicles labeled during spontaneous network activity are repositioned in AZ proximity for preferential reuse. The inclusion of NbLumSyt1-ALFA-tag: NbALFA-Gold in active endocytic processes was captured in tomographic subvolumes containing labeled endocytic intermediates and newly formed vesicles in proximity to the plasma membrane (Fig. 2r-s). On average, recycling CCSV^G^s exhibited a slightly larger diameter compared to unlabeled CCSVs (Fig. 2t-u) independent of active zone proximity (Fig. 2v). The relatively low percentage of recycling vesicles per tomogram labeled by NbLumSyt1-ALFA-tag: NbALFA-Gold complex during spontaneous activity (mean ± SEM = 7.88 ± 2.915%)(Fig. 2w) is consistent with previously published estimates obtained using alternative strategies. Collectively, these ultrastructural analyses indicated that the NbLumSyt1-ALFA-tag: NbALFA-Gold complex labels actively recycling SVs, providing a robust framework for investigating the mechanisms of vesicle trafficking at the synapse.

### Exo-endocytic cycling of SVs monitored with novel NbLumSyt1 reporters

To test whether labeling with NbLumSyt1 affects synaptic function, we analyzed SV exo- and endocytosis in living neurons. To this end, we created fusion proteins in which NbLumSyt1 was fused to the GFP variant e-pHluorin [22] (pHluorin) or mOrange, both of which are quenched in the acidic interior of the SV but rendered fluorescent upon SV fusion at the plasma membrane.

**Fig. 3:**
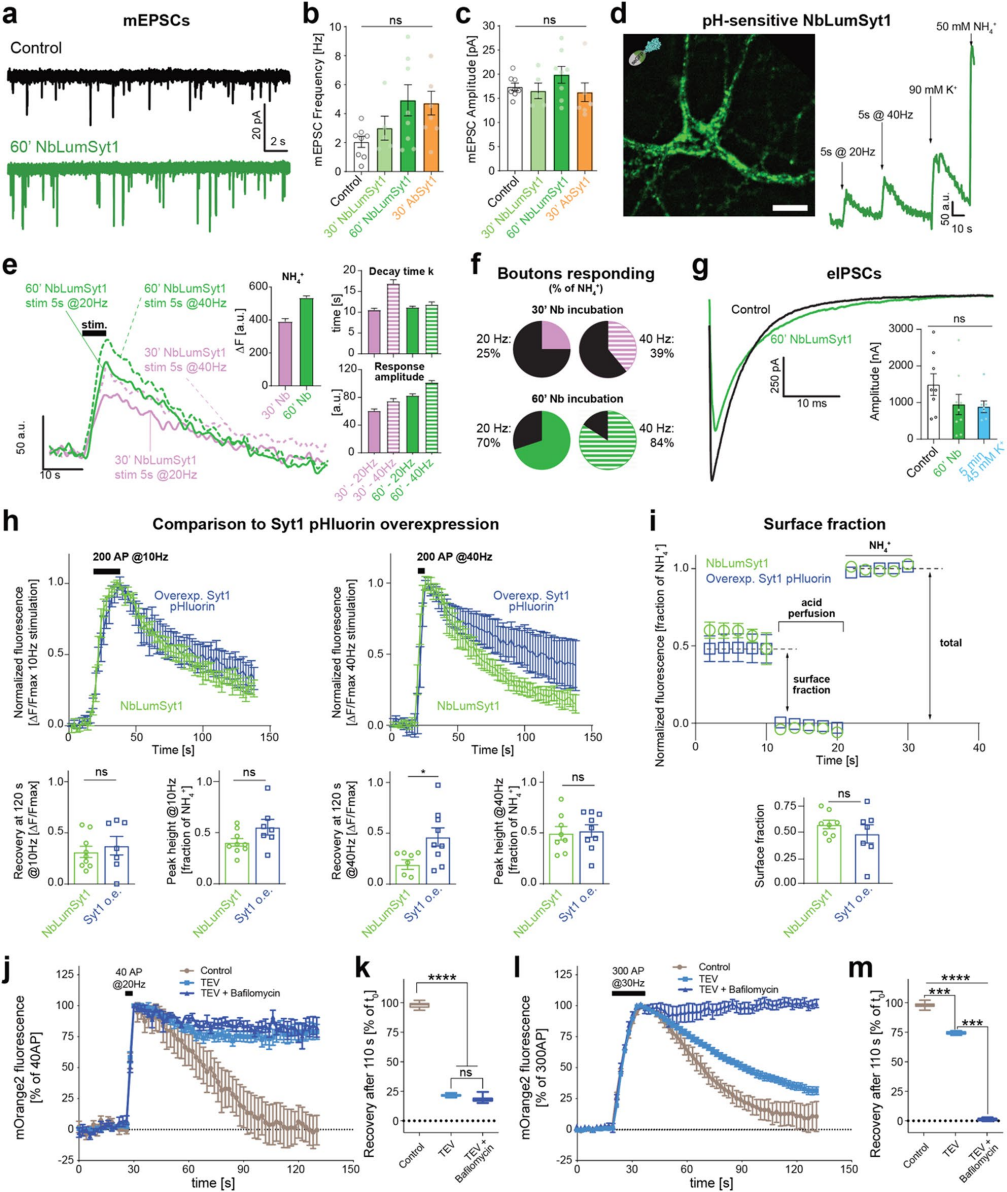
NbLumSyt1 is a minimally invasive tool for measuring synaptic vesicle exo-endocytosis. **a-c**) Miniature excitatory postsynaptic current (mEPSC) measurements in control (no nanobody or antibody treatment) and treated neurons with different loading times, either with NbLumSyt1 directly fused with pHluorin (NbLumSyt1-pHluorin) or with a commercial Syt1-luminal antibody (SySy 604.2). **d**) Live imaging of NbLumSyt1-pHluorin recycling. The fluorescence increase indicated nanobody uptake upon stimulation, followed by florescence decay due to endocytosis of the nanobody. Treatment with ammonium chloride (NH_4_^+^) revealed the total pool of labeled vesicles. Scale bar: 10 μm. **e**) Example traces with decay time constants (tau) and amplitudes upon electrical stimulation of cells labeled with NbLumSyt1-pHluorin. Statistical significance was assessed by one-way ANOVA followed by Tukey’s multiple comparisons test. Although there is a trend, no significant differences were found. **f**) Percentage of boutons responding to different electrical stimuli (calculated as a percentage of the total pool of SVs, as defined by NH_4_^+^ treatment). **g**) Evoked inhibitory postsynaptic currents (eIPSCs), which are used to study the drive of GABAergic neurotransmission. Control neurons were stimulated with 45 mM KCl for 5 min without the nanobody. Inset: Quantification of eIPSC amplitude. **h**) Comparison of the effects of NbLumSyt1-pHluorin on neurons overexpressing Syt1 pHluorin. The overexpression of Syt1-pHluorin causes slower endocytosis at higher (40 Hz) stimulation frequency. **i**) Evaluation of the surface fraction by perfusion with acidic buffer (pH 5). Ammonium chloride perfusion at the end of the experiment was used to reveal the total population of molecules (used for normalization). The surface fraction was unchanged at our Syt1-pHluorin expression levels. **j**-m) The fluorescence increase and recovery in neurons loaded with NbLumSyt1-TEV-mOrange2, incubated with the TEV protease, and then field-stimulated either for 2 s at 20 Hz (**j**) or for 10 s at 30 Hz (l) in the presence or absence of the reacidification blocker bafilomycin. Under control conditions, as expected, fluorescence is quenched and returns to baseline after endocytosis due to acidification of the endocytosed Syt1 pools. After mild stimulation (**j**, recovery at 110 s quantified in **k**), the fluorescence in TEV-cleaved neurons does not recover; but in contrast, after more pronounced stimulation, fluorescence quenching is observed (**l**, recovery at 110 s quantified in **m**). The graphs summarize the results of 3 independent experiments. The error bars indicate the means ± SEMs; ns, not significant, * *p* < 0.05, *** *p* < 0.001,**** *p* < 0.0001

To ensure minimal perturbation of neuronal activity from NbLumSyt1 labeling, miniature excitatory postsynaptic currents (mEPSCs) were recorded after loading neurons with either NbLumSyt1-pHluorin or a commercial Syt1 luminal antibody and compared to those of controls. Prolonged loading (60 min) resulted in a small, nonsignificant increase in mEPSC frequency. In any case, to avoid unnecessary alterations in all subsequent experiments, we decided to use a 30-min loading, since the signal was sufficient, and no changes in activity were observable (Fig. 3a-c). Nonetheless, we suggest general prudence, as Syt1 luminal domain antibodies have previously been associated with partial loss-of-function effects [23].

Live imaging of NbLumSyt1-pHluorin recycling revealed dynamic changes in fluorescence upon electrical stimulation. A major increase in fluorescence was observed by exocytotic exposure of the previously internalized nanobody to the extracellular surface, which scales with the stimulation intensity, followed by rapid decay due to endocytosis and reacidification. Ammonium chloride treatment, which neutralizes the pH across all compartments, including the SVs, allowed us to estimate the total pool of labeled vesicles (Fig. 3d).

Analysis of fluorescence decay kinetics demonstrated consistent tau values across conditions, supporting the tool’s utility in quantifying vesicle dynamics (Fig. 3e). The high proportion of boutons responding to increasing electrical stimuli further confirmed the reliability of this labeling method (Fig. 3f). Moreover, no significant differences in evoked inhibitory postsynaptic currents (eIPSCs), a measure of GABAergic neurotransmission, were observed, indicating that nanobody labeling does not measurably perturb synaptic function (Fig. 3g).

Comparisons of NbLumSyt1-pHluorin-labeled neurons with those overexpressing Syt1-pHluorin highlighted key differences: Syt1-pHluorin overexpression slowed endocytosis during high-frequency (40 Hz) stimulation (Fig. 3h). Perfusion with acidic buffer, which selectively quenches surface-exposed pHluorin, revealed that the size of the plasma membrane pool after exocytosis was unchanged compared with that of neurons overexpressing Syt1-pHluorin. (Fig. 3i) Together, the data show that labeling of neurons with NbLumSyt1 does not perturb SV recycling kinetics.

To differentiate between distinct surface-exposed and internalized pools of NbLumSyt1-labeled Syt1, we created modified fusion proteins in which NbLumSyt1 was connected to reporter domains via a linker containing a cleavage site for the tobacco etch virus (TEV) protease (i.e., NbLumSyt1-TEV-pHluorin, NbLumSyt1-TEV-mOrange). Efficient cleavage was observed when the products were analyzed by SDS-PAGE after TEV treatment (Supplementary Fig. 3a). When neurons were preincubated with the mOrange2 variant and then treated with the TEV-protease to remove the fluorescent surface-exposed pool, cleavage was completed within a few minutes (Supplementary Fig. 3b), similar to that of the purified proteins (Supplementary Fig. 3c).

As previously reported [24], this approach allows for differentiating between freshly exocytosed and longer-lingering surface pools of Syt1. To this end, neurons preloaded with NbLumSyt1-TEV-mOrange2 were treated with TEV-protease to remove all preexisting labels exposed on the surface under resting conditions. After washout of the protease, the neurons were moderately stimulated to selectively exocytose the readily releasable SVs (Fig. 3j, k). Note that the control cells were loaded with NbLumSyt1-TEV-mOrange2 but not treated with TEV. As expected, the fluorescence intensity increased rapidly since the newly exposed intravesicular sensors were protected during protease pretreatment. After the end of the stimulus training, a rapid decay was observed in the untreated samples. However, the degree of decay was much lower in the TEV-pretreated condition, confirming that upon mild stimulation, the preexisting plasma membrane pool (rendered nonfluorescent by TEV pretreatment) is preferentially internalized by compensatory endocytosis [24]. During longer stimulation trains a much greater proportion of SVs underwent exocytosis followed by clear endocytosis. In this stimulation paradigm this revealed both the exo-endocytosis of vesicles and that the readily retrievable pool was limited (Fig. 3l, m). Notably, in the presence of bafilomycin, an inhibitor of vesicular V-ATPase, which was used here as a control, no decay of the fluorescence signal was observed during endocytosis since reacidification was inhibited [25]. The persistence of a fluorescence plateau in the presence of bafilomycin argues against rapid reacidification-dependent retrieval mechanisms (such as ultrafast endocytosis or kiss-and-run) contributing substantially to this signal. These data likely support the interpretation that mild stimulation preferentially mobilizes a vesicle subset that is largely distinct from the pool retrieved by compensatory endocytosis. Instead, stronger stimulation progressively recruits vesicles that subsequently undergo endocytosis and reacidification. This interpretation is well in line with previous work demonstrating functional segregation between exocytosed and endocytosed vesicle pools under different stimulation regimes [26].

### Resting pH estimations with GFP-based nanobody reporters

**Fig. 4:**
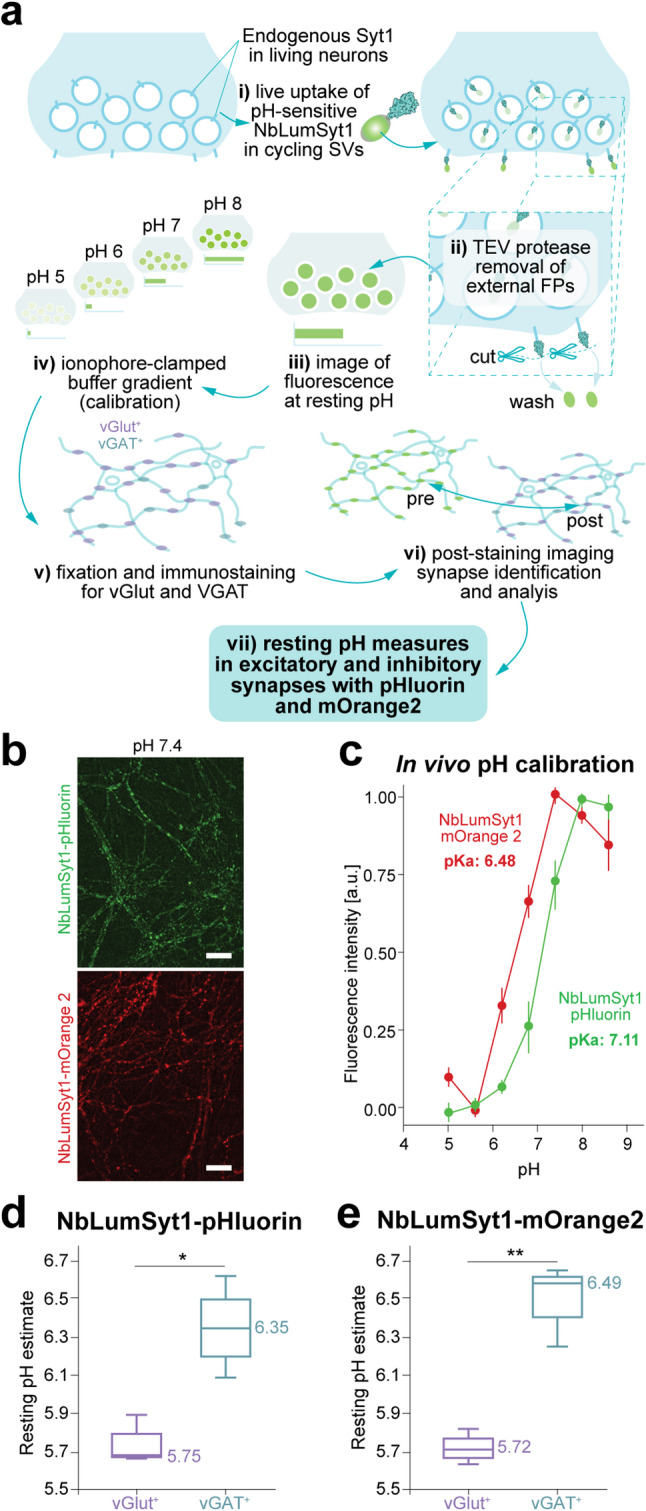
Estimation of resting pH using NbLumSyt1-pHlourin and NbLumSyt1-mOrange2 probes in rat hippocampal neurons. **a**) Scheme of the experimental design for estimation of the resting pH of SVs. The resting pH of either excitatory or inhibitory boutons was estimated similarly to what was previously described [27], albeit with minor modifications (see methods). Neurons were labeled with either NbLumSyt1-pHluorin or NbLumSyt1-mOrange2 in both cases containing a TEV recognition site for the TEV protease. **b**) Images of neurons in pH 7.4 buffer after treatment with TEV protease to remove the pH-sensitive fluorescent proteins from the surface pool (see also Supplementary Fig. 3 a-c). Scale bar 10 μm. **c**) Quantification of fluorescence intensity of NbLumSyt1-pHluorin or NbLumSyt1-mOrange2 in buffers adjusted to certain pHs and the estimated pKa values (see also Supplementary Fig. 3 d-f for pKa estimation from in vitro data). **d**,** e**) The resting pH values calculated from the calibration curves based on estimated pKa values (see methods). The values obtained by the two probes were similar and revealed a significant difference between excitatory and inhibitory boutons (NbLumSyt1-pHluorin (**d**), NbLumSyt1-mOrange2 (**e**)). Unpaired Student’s t test, *N* = 3. * *p* ≤ 0.05, ** *p* ≤ 0.01. Note that the numbers in the graphs indicate average values

We utilized the NbLumSyt1-pHluorin and -mOrange2 constructs to revisit some of the previous work on the resting pH of SVs in synaptic boutons [27]. mOrange2 has a lower pK value than pHluorin does, thus allowing the measurement range to be extended toward lower pH values, such as those found in SVs [27] (Supplementary Fig. 3). To this end, the neurons were allowed to internalize the probes, followed by TEV cleavage to remove all the extracellular labels. After fluorescence was recorded, boutons were pH-clamped at defined pH values for calibration, fixed, and immunostained for vesicular glutamate transporters (VGLUT) and GABA transporters (vGAT) to differentiate between excitatory and inhibitory boutons (Fig. 4). Neurons were treated with buffers containing ionophores that were adjusted to increasing pH values, and images were acquired at different pH steps. At the end of the experiment, the sample was fixed and immunostained with antibodies specific for VGLUT and VGAT, and the same region was located again (post hoc) and reimaged. The images were aligned and the fluorescence intensity was quantified, from which the pKa values of the two probes were calculated (Fig. 4b, c). The estimated resting pH values (see methods) showed significant differences between glutamatergic and GABAergic neurons, where glutamatergic neurons showing a lower resting pH than GABAergic neurons did (Fig. 4d, e), in agreement with previous results [27].

### Live-cell proteomic mapping of Syt1 surface interactions via NbLumSyt1-APEX2

To perform live-cell mapping of Syt1 surface interactions with other proteins and potentially identify novel Syt1 interaction partners/modulators, we developed a version of the nanobody conjugated to APEX2 [28]. This enzyme enables efficient biotinylation of proteins in close proximity to a protein of interest, in our case Syt1, during live-cell uptake.

**Fig. 5:**
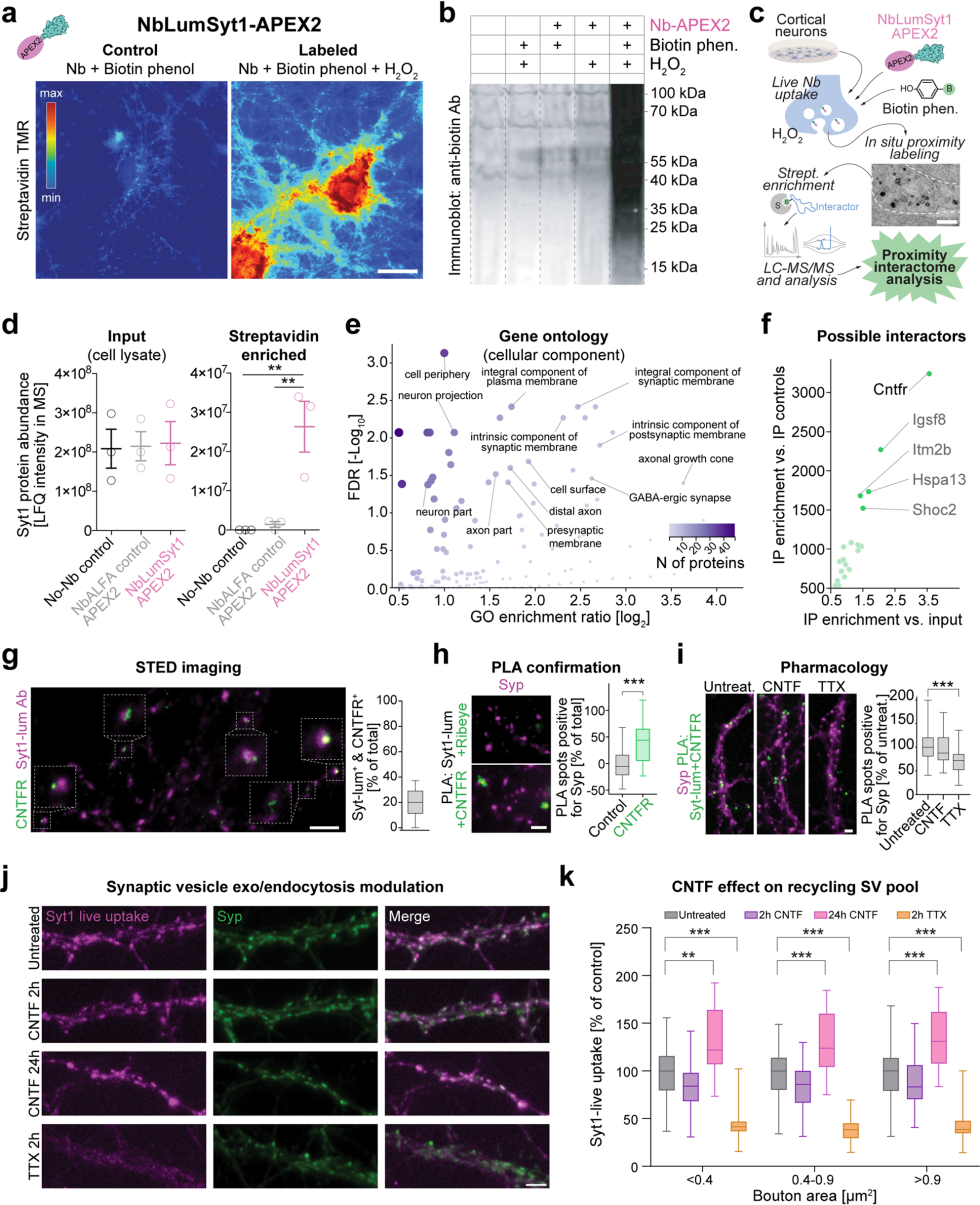
Syt1 is found in close proximity to the ciliary neurotrophic factor receptor (Cntfr) and regulates synaptic vesicle dynamics. **a-c**) NbLumSyt1-APEX2 allows efficient biotinylation of proteins in the proximity of Syt1 upon live uptake in hippocampal neurons to facilitate live-cell proteomic mapping. Representative images of neurons upon uptake of NbLumSyt1-APEX2, where biotinylated proteins are revealed with fluorescent streptavidin **(a)**. In the absence of H_2_O_2_, only a few endogenous biotinylated proteins are observable. In the presence of all the components, the reaction occurred efficiently, as revealed in western blot analysis of labeled neurons (**b**). To identify the interactors of Syt1, in situ proximity labeling was performed with NbLumSyt1-APEX2 (**c**). The electron microscopy image in the scheme is an example of the labeled vesicles, as revealed upon photoconverting 3,3’-diaminobenzidine (DAB) into a stable, electron microscopically visible dark product. **d**) Protein intensities measured with LC‒MS/MS at the input and upon enrichment of the biotinylated proteins. Two controls were used: neurons without nanobodies or neurons where an unrelated nanobody (anti-ALFA-Nb) was provided in the medium. Note that since the primary neurons do not express the ALFA tag, this control will reveal the effect of the unspecific biotinylation of the membranes occurring during the labeling period. Note that Syt1, as expected, is efficiently biotinylated and enriched upon IP with streptavidin beads. See methods for details concerning the experiments and analyses. **e**) Summary of the gene ontologies (GOs; cellular components) for the proteins biotinylated upon live uptake of NbLumSyt1-APEX2 (for a detailed list, see Supplementary Table 1). As expected, synaptic components and membrane GO terms were overrepresented. **f**) Possible interactors identified via live-cell proteomic mapping and enrichment vs. input and vs. IP control. Cntfr was found to be the most enriched candidate, together with other proteins that could be studied in future works. **g**) Super-resolution stimulation emission depletion (STED) imaging reveals that ~ 20% of boutons labeled with live uptake are also positive for Cntfr. In this case, for cross-validation purposes, live uptake was performed with the 604.2 Syt1-luminal antibody. **h**) Proximity ligation assay (in situ PLA) using antibodies against the luminal portion of Syt1 and anti-Cntfr confirmed the close proximity of these two proteins. A primary antibody against a protein not expressed in hippocampal neurons (Ribeye) was used as the negative control. **i**) Blocking the network activity of primary hippocampal neurons with tetrodotoxin (TTX) for 2 h decreases the in situ PLA signal between Syt1 and Cntfr. Stimulation with the ligand of Cntfr (Cntf; 8 nM) for 2 h does not change the PLA signal between Syt1 and Cntfr. **j**,** k**) Stimulation of neurons with Cntf for 24 h increases SV exo-endocytosis. Scale bars: 10 μm in **a**; 500 nm in **c**; 5 μm in **g-j**. The error bars indicate the means ± SEMs for panel d, and the 5th or 95th percentile for box plots; ** *p* < 0.01; *** *p* < 0.001

Representative experiments with NbLumSyt1-APEX2 show that biotinylation occurs only when all reaction components, including H_2_O_2_, are present, as revealed by fluorescent streptavidin staining (Fig. 5a). WB analysis confirmed robust biotinylation under these conditions (Fig. 5b). Moreover, in situ proximity labeling performed with NbLumSyt1-APEX2, coupled with deposition of electron-dense material visible via EM, revealed labeled SVs (Fig. 5c). Having established efficient protein biotinylation and targeting, we performed protein identification following labeling and streptavidin bead pull-down. Proteome analysis by liquid chromatography followed by mass spectrometry (LC‒MS/MS) revealed enrichment of Syt1 (positive control) and other interactors (Supplementary Table 1**)**. Importantly, label-free quantification (LFQ) intensities represent relative, normalized abundance estimates and do not reflect absolute recovery or labeling efficiency; therefore, comparisons between input and IP values cannot be interpreted as a fraction of total Syt1 captured. Controls using neurons without nanobody uptake or labeled with NbALFA-APEX2 demonstrated minimal nonspecific biotinylation (Fig. 5d).

Gene Ontology (GO) analysis of the candidates enriched in the NbLumSyt1-APEX2 samples clearly revealed synaptic and membrane-associated components, which was consistent with the expected localization of Syt1 interactors (Fig. 5e). Interestingly, among the identified interactors, the ciliary neurotrophic factor receptor (Cntfr) was the most enriched hit, alongside additional proteins that may be the subject of future studies (Fig. 5f). Cntfr is a cell surface receptor involved in neuroprotection and synaptic maintenance [29], whose potential partnership with Syt1 could regulate SV dynamics, specifically changing vesicle release or recycling efficiency at synaptic terminals. Another interesting candidate, integral membrane protein 2B (Itm2b), stands out since it plays a role in vesicle trafficking, is linked to neurodegenerative diseases involving synaptic dysfunction, and may regulate SV recycling and neurotransmitter release [30].

To further characterize the possible local interaction of Syt1 and Cntfr, we performed super-resolution stimulation emission depletion (STED) imaging on neurons costained for these two proteins and used an antibody against the Syt1-luminal domain (Syt1-lum Ab, clone 604.2) for cross-validation purposes. These experiments revealed that ~ 20% of the synaptic boutons positive for Cntfr were also positive for live uptake of Syt1, confirming that the two molecules are indeed found in close proximity (Fig. 5g). To confirm their direct local interaction, a proximity ligation assay (PLA) with Syt1-lum and Cntfr was established and revealed close proximity, with a minimal signal detected when a nonneuronal protein was used as a negative control (Fig. 5h). To further test possible functional crosstalk between the two molecules, we performed experiments in which the activity regimens of our neurons were modulated. Blockade of network activity with tetrodotoxin (TTX) decreased the PLA signal between Syt1 and Cntfr, indicating that inhibition of neuronal activity leads to a decrease in the interaction of these two proteins. Importantly, direct stimulation with the Cntfr natural ligand Cntf for 2 h did not alter the PLA signal, indicating that Cntf binding does not significantly affect the proximity of Syt1-Cntfr at short time points (Fig. 5i). However, prolonged stimulation with Cntf (24 h) led to an increase in SV exo-endocytosis, revealing a role for Cntfr in regulating synaptic activity (Fig. 5j-k).

### Nanoscale organization of endogenous surface-stranded Syt1 via NbLumSyt1-Halo reveals two populations of molecules with distinct dynamics

**Fig. 6:**
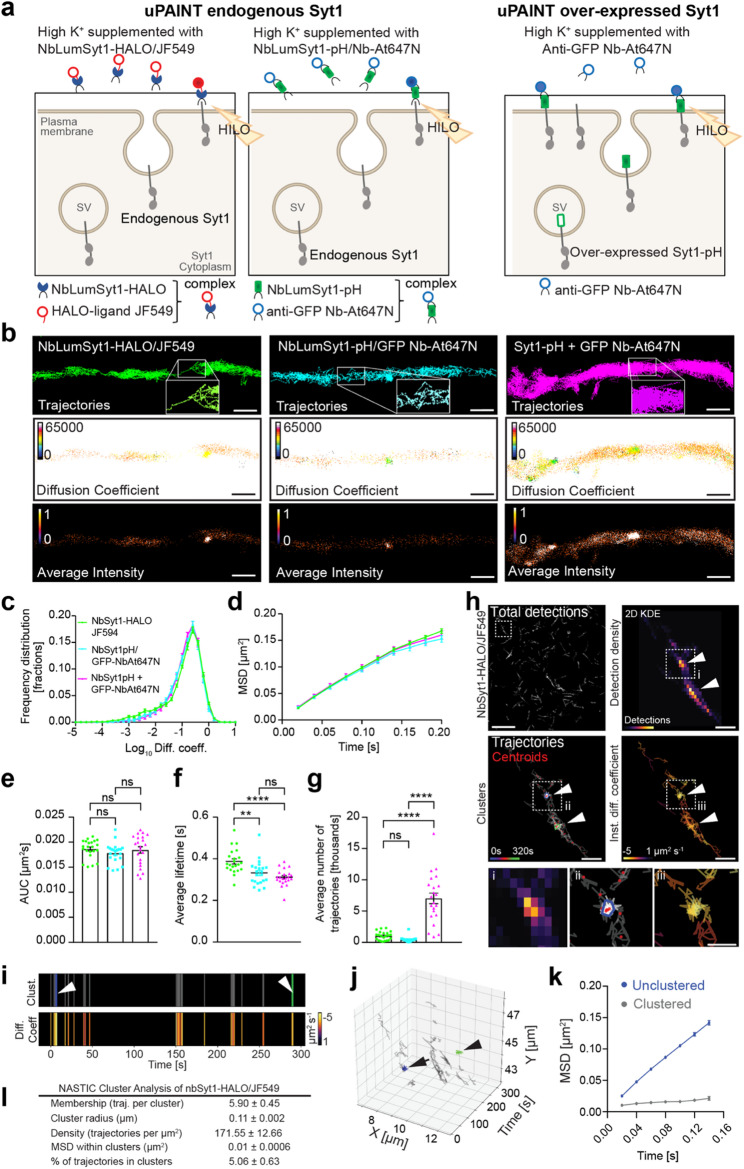
Live-cell single-molecule imaging of endogenous Syt1 reveals two populations of molecules whose displacement dynamics differ. **a**) Scheme of single fluorescent molecule detection in live hippocampal neurons via uPAINT. To detect and track single molecules, cells were incubated with complexes conjugated prior to imaging the NbLumSyt1-Halo-Tag with the Halo-ligand JF549 or the NbLumSyt1-pHluorin with the anti-GFP Nb-At647N (NbLumSyt1-pH/Nb-At647N). Individual trajectories correspond to probe-binding events and subsequent lateral diffusion of surface-exposed Syt1 molecules and do not represent the full exocytosis–endocytosis cycle of individual synaptic vesicles. b) Super-resolved images of single-molecule trajectories, diffusion coefficients, and average intensities in hippocampal neurons over 16,000 frames. The color scale in the diffusion coefficient map ranges from 0 to 1, corresponding to Log10 diffusion coefficient detections, and the colder colors in the scale indicate lower mobility. The average intensity map represents localization densities as arbitrary units, with warmer colors indicating higher detection densities. c-g) Quantification of the parameters under different conditions. The lifetime decreases with increasing complex size, and the overexpression of Syt1 results in a significantly greater number of trajectories than do the endogenous Syt1 trajectories. h) Representative image showing detection of the NbLumSyt1-Halo/JF549 complex from a 320 s acquisition at 50 Hz by imaging with uPAINT in cultured hippocampal neurons. The arrowheads indicate two separate NbLumSyt1-Halo/JF549 clusters, and the boxed areas (i-iii) are magnified. The resulting NASTIC analysis images of 2D kernel density estimation (KDE) of detections with single-molecule trajectories, centroids, clusters, and instantaneous diffusion coefficients (log10) from the boxed region are shown magnified in the boxed regions. The trajectories are shown in gray, with each trajectory segment representing the displacement of the detected molecule in 0.02 s. The centroid of each trajectory is indicated with a red dot. The colored convex hulls indicate the extent of the detections associated with clustered trajectories as determined by NASTIC. i) The top panel shows the 2D temporal clustering NbLumSyt1-Halo/JF549 (arrow and arrowhead point to the timeframe of blue and green clusters shown in h) over 320 s, and the lower panel shows the respective diffusion coefficient mobility of the trajectories, indicating a lower mobility of NbLumSyt1-Halo/JF549 within clusters than when unclustered. j) 3D representation of the spatiotemporal clustering of NbLumSyt1-Halo/JF549, where the blue and green clusters from c and f are indicated. k) The graph shows the quantification of the mean square displacement (µm2 s− 1) of NbLumSyt1-Halo/JF549 within clusters and outside of the clusters (unclustered). MSD curves measure the average mobility with respect to time of all the trajectories observed. l) Biophysical properties of NbLumSyt1-Halo/JF549 clusters (n = 10 neurons). The numbers represent average values ± SEMs. Scale bars: 1 μm. Statistical analysis of normally distributed data was performed via one-way ANOVA for multiple comparisons in g, and for nonnormally distributed data, one-way ANOVA with the Kruskal‒Wallis test for multiple comparisons was used. ** p < 0.01, **** p < 0.0001.

Nanobodies offer several advantages for diffraction-unlimited imaging [31]. This has recently allowed the characterization of the surface clustering of Syt1 following exocytosis and established a novel role in coordinating the binding and SV targeting of botulinum neurotoxin type A [32]. To track endogenous Syt1 behavior under live conditions following exocytosis and avoid perturbations due to Syt1 overexpression, we performed single-molecule imaging of surface endogenous Syt1 in live hippocampal neurons with universal point accumulation for imaging in nanoscale topography (uPAINT) [33, 34]. Labeling of Syt1 was achieved by incubating neurons with preconjugated complexes: either NbLumSyt1-HALO bound to the Halo-ligand JF549 or NbLumSyt1-pHluorin precomplexed with the anti-GFP nanobody conjugated to Atto647N. Imaging was performed via highly inclined and laminated optical microscopy (HILO) to increase signal-to-noise discrimination. For comparison, neurons overexpressing Syt1-pHluorin were imaged under identical conditions using the anti-GFP nanobody Atto647N (Fig. 6a) [35]. This experimental setup allowed the detection and tracking of single endogenously and exogenously expressed Syt1 molecules with high temporal resolution (20 ms), providing super-resolved images of single-molecule trajectories and diffusion coefficients (Fig. 6b). The map of diffusion coefficients showed heterogeneous mobility, with colder colors representing lower mobility, whereas average intensity maps, which indicated localization densities, showed distinct regions of lower (cold colors) and higher detection densities (white color; Fig. 6b). To avoid any misunderstandings, uPAINT reports individual probe-binding events to surface-exposed Syt1 molecules, as well as their lateral mobility. Therefore, individual trajectories do not report the exo-endocytosis cycle of single synaptic vesicles.

The quantification of single-molecule mobilities via the three labeling techniques revealed similar frequency distributions of diffusion coefficients (log_10_D), mean square displacement (MSD) and area under the MSD curve (AUC) values (Fig. 6c-e), whereas the fluorescence lifetimes of the labeled complexes decreased with increasing number of labeling tools, which may reflect certain limitations of bulkier affinity tools (Fig. 6f). As expected, neurons overexpressing Syt1-pHluorin presented a significantly greater number of trajectories than those labeled with endogenous Syt1. Single-molecule detection and tracking of NbLumSyt1-HALO/JF549 on the membrane of hippocampal neurons revealed distinct confinement areas (Fig. 6h; total detection). The spatiotemporal clustering [36] of endogenous Syt1 molecules was further investigated via two-dimensional kernel density estimation (2D KDE) to identify trajectories associated with Syt1^+^ clusters (Fig. 6h). The Syt1^+^ trajectories detected in clusters displayed lower mobility than the unclustered molecules did, as evidenced by their reduced diffusion coefficients and confinements (Fig. 6i). Visualization of the spatiotemporal clustering [36] of the tracked Syt1^+^ surface molecules revealed multiple immobilization zones detected concomitantly with more mobile trajectories over time (Fig. 6j), as recently described [37, 38]. Quantifying the MSD of Syt1 via molecular dynamics in these spatiotemporal clusters revealed significantly reduced mobility (Fig. 6k). Some areas presented clusters characterized by lower estimated diffusion coefficients associated with reduced mobility. In contrast, the unclustered molecules had greater mobility (Fig. 6k), revealing two populations of surface molecules, one probably engaged in more complex interactions (clustered) and one less restrained (unclustered). The quantification of the biophysical properties of the NbLumSyt1-Halo/JF549 nanoclusters is shown in Fig. 6l. This nanoscale analysis clearly identified two subpopulations of Syt1 molecules with distinct cluster associations on the basis of their displacement dynamics, providing insights into the functional nano-organization of SV components following exocytosis on the plasma membrane. The functional significance of these surface clusters has recently been revealed, as Syt1, polysialoganglioside and SV2A form tripartite surface nanoclusters that act as receptors for botulinum toxin type A ^32^. Combined with our demonstration that Syt1 overexpression alters the rate of endocytosis at high stimulation rates, our data suggest that altering Syt1 synaptic abundance impacts the maintenance of synaptic function.

To further investigate the use of NbLumSyt1 in another live-imaging diffraction-unlimited modality, we used MoNaLISA (Molecular Nanoscale Live Imaging with Sectioning Ability) imaging [39], a parallelized RESOLFT approach tailored to the dynamic organization of molecules in living cells. In this imaging modality, SVs were live-labeled with NbLumSyt1-rsEGFP (N205S) in neurons. Through the combination of extended light patterns, MoNaLISA enhanced the spatial resolution to nanoscale clusters as small as 65 nm with an intercluster distance of approximately 130 nm over an extended field of view within a frame time of 2 s (Supplementary Fig. 4a). Comparisons between the wide-field and MoNaLISA images illustrated the resolution gained (Supplementary Fig. 4b, c). Time-lapse imaging of the NbLumSyt1-rsEGFP(N205S)-labeled vesicles revealed marked structural rearrangements, even at 1 min of separation (Supplementary Fig. 4d, e). The quantification of single clusters revealed dynamic changes in their size, ellipticity, and intensity (Supplementary Fig. 5f). These findings suggest that SV organization rapidly changes at the nanoscale. Furthermore, dual-color imaging of NbLumSyt1-rsEGFP(N205S) together with the inhibitory synapse marker nanobody anti-vGAT-Cy3 revealed enrichment of labeled clusters at inhibitory synapses (Supplementary Fig. 4g-i). Compared with their excitatory counterparts, inhibitory synaptic clusters were larger in area, more elliptical in shape and had higher mean fluorescence intensity. Overall, these features indicate denser and more organized vesicle distribution at inhibitory synapses, which is consistent with functional specialization in these synaptic domains [40], and highlight the usefulness of live-imaging diffraction-unlimited modalities.

### Translational application of NbLumSyt1 for real-time monitoring of synaptic vesicle recycling in human neuronal systems

**Fig. 7:**
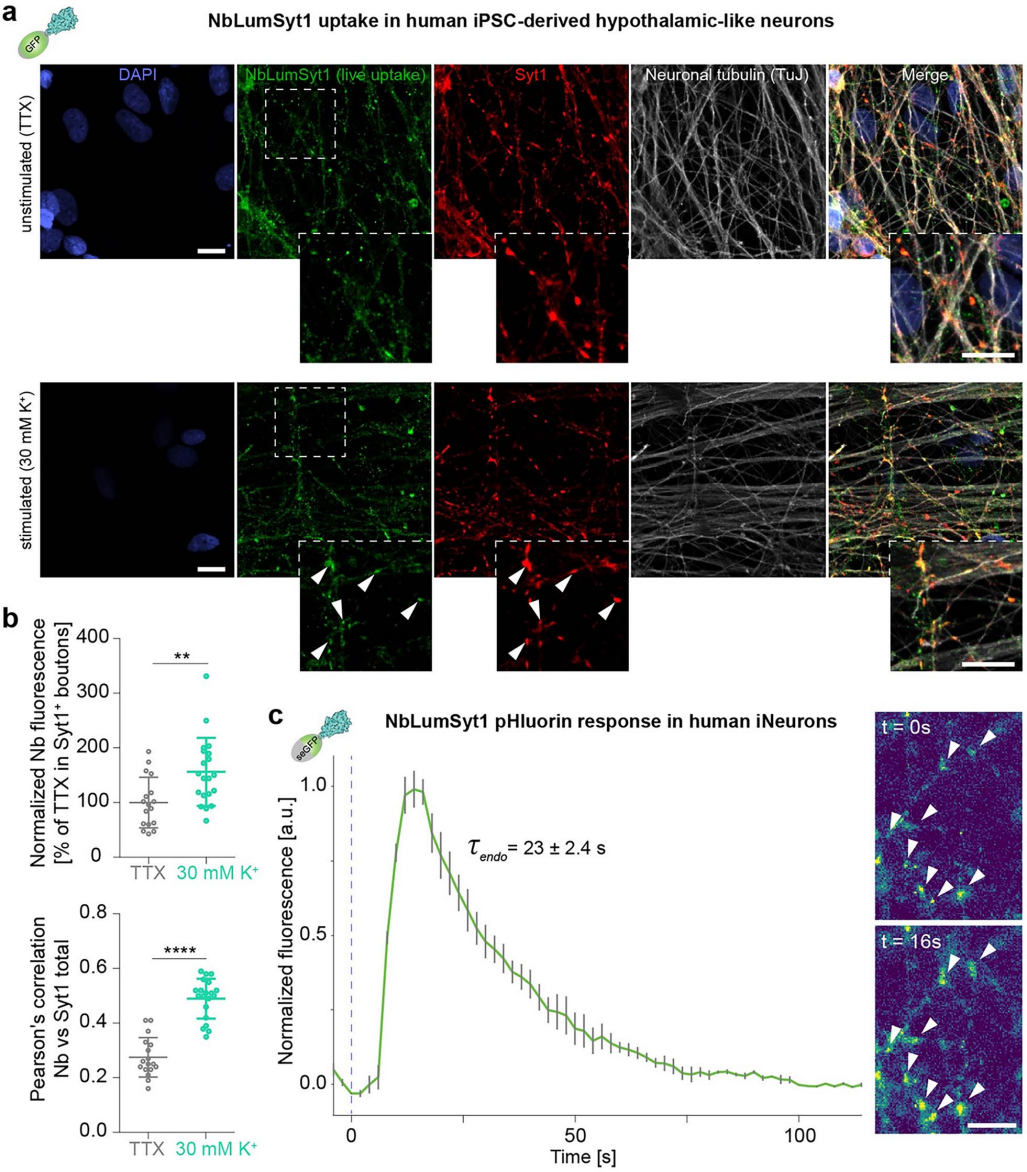
NbLumSyt1 specifically binds human Syt1 and can be used for recycling assays in human neurons. **a**) Human iPSC-derived hypothalamic-like neurons were stained live with NbLumSyt1 and, after recycling, fixed and immunoassayed against endogenous Syt1 and the neuron-specific betaIII tubulin marker (TuJ). The cells were pretreated with TTX or K^+^ during labeling. **b**) Quantification indicates that increased stimulation during labeling increases both the labeling level (upper panel) and the correlation between luminal Syt1 and total Syt1 (lower panel). Unpaired Student’s t test; the error bars indicate the means ± SEMs; ** *p* < 0.01, **** *p* < 0.0001. **c**) Synaptic vesicle recycling measured in iNeurons monitored with the pH-sensitive version of NbLumSyt1. (Left) Averaged normalized pHluorin fluorescence traces from iNeurons (6–8 weeks in culture, *N* = 3) stimulated with 200 action potentials (40 Hz, 5 s) at physiological temperature (37 °C). The blue segmented line indicates the beginning of the stimulation. (Right) Representative images showing the sensor intensity before (t = 0 s) and directly after (t = 16 s) electrical stimulation. Scale bars: 10 μm in **a**; 5 μm in **c**

To demonstrate the translational potential of NbLumSyt1, we characterized the performance of our nanobinding tools in human neurons. Live staining of human induced pluripotent stem cells (iPSCs)-derived hypothalamic-like neurons (hypothalamic-iNeurons) with NbLumSyt1 followed by fixation and subsequent immunoassay for endogenous Syt1 and the neuronal marker beta-III-tubulin (TuJ) confirmed the neuronal identity and synaptic pattern of these cells. Pretreatment with TTX strongly reduced activity-dependent labeling of NbLumSyt1, while pretreatment with increased K^+^ concentration increased the labeling, confirming that most labeling was driven by neuronal activity. A basal level of staining persisted, reflecting surface binding and spontaneous vesicle recycling occurring independently of evoked activity (Fig. 7a). Quantification revealed that the overall intensity of NbLumSyt1 staining correlated with neuronal activity. A positive correlation between the luminal and total Syt1 staining levels further confirmed the dependence of the probe on activity under stimulation conditions. In agreement with the sensitivity to physiological synaptic activity, NbLumSyt1 staining levels were significantly reduced when the cells were treated with TTX (Fig. 7b).

We used NbLumSyt1-pHluorin to label SVs directly in human fibroblast differentiated to cortical neurons (cortical-iNeurons) [41] and measure SV recycling. Stimulation with 200 action potentials at 40 Hz elicited a dynamic fluorescence response that captured the temporal progression of exocytosis followed by endocytosis at physiological temperature. Averaged fluorescence traces demonstrated the reliability of NbLumSyt1-pHluorin in quantifying SV dynamics in real time. Representative images before and after stimulation show the resolution and sensitivity of the tool in tracking these processes at the synaptic level (Fig. 7c). These findings highlight the usefulness of NbLumSyt1 as a reliable molecular probe for studying SV recycling mechanisms in human neuronal systems.

## Discussion

The development of NbLumSyt1 represents a significant advance within the synaptic pathophysiology toolkit, allowing flexibility and future applications for human studies to interrogate SV recycling and synaptic physiology in a clinically relevant context. This nanobody-based approach targets the luminal domain of Syt1, a central regulator of SV exocytosis and endocytosis, in minimally invasive and versatile manner, while its effective specificity depends on the experimental context (see below for limitations and recommended controls). Although Syt1 has long been noted in previous studies to play a crucial role in calcium-dependent neurotransmission and SV recycling [7], restrictions in the available arsenal of tools have hampered real-time and high-resolution visualization of these processes, especially in human neurons. NbLumSyt1 overcomes these challenges by combining fluorescence-based live imaging, ultrastructural studies, and proteomic analyses in a future clinically oriented study of synaptic physiology.

The live-cell imaging applications of NbLumSyt1, especially when coupled with either HALO or pHluorin tags for the acquisition of spatiotemporal dynamics in the process of SV recycling, highlight the flexibility of this tool. These experiments revealed the spatial distributions of SV pools and their dynamics during recycling. Our results align well with the previously reported heterogeneity and dynamic nature of SV pools [10, 37, 38]. Notably, the high signal-to-noise ratio achieved with NbLumSyt1-HALO and the multiplexing capabilities provided by an extensive palette of available HALO ligands [42] highlight its potential to detect subtle changes in SV behavior under physiological and pathological conditions. Furthermore, fluorescence-based pHluorin assays offer simple, convenient and minimally invasive means to estimate intravesicular pH, an important parameter controlling neurotransmitter loading and release [22]. These tools constitute an essential step forward in bridging the long-standing gap in the measurement of SV recycling dynamics without disrupting synaptic function [43]. While previous studies have debated the role of the luminal region of Syt1 in glycosylation and vesicle targeting [44, 45], our work seems to suggest that the nanobody has no direct influence on these processes. Although no functional effects were observed in our study under our conditions, previous observations with antibodies [23] suggest that potential effects should be evaluated on a case-by-case basis.

Ultrastructural studies of NbLumSyt1-ALFA-tag further validated its specificity and functional relevance. The EM labeling of active SV recycling pathways has shown preferential vesicle recruitment with distinct molecular signatures in support of selective vesicle reuse during synaptic activity. This study thus offers an additional tool to support nanoscale structural analysis and functional investigations by combining advanced EM with nanobody-based labeling modalities for nanogold staining.

Another critical approach is the development of proteomic mapping by employing NbLumSyt1-APEX2 to enable the probing of novel interacting proteins involved in regulating SV dynamics. The interaction of Syt1 with Cntfr identifies a previously unacknowledged molecular link between synaptic vesicle cycling and receptor-related signaling, providing a concrete biological example of the types of questions that flexible tools such as the NbLumSyt1 enables. Future experiments are needed to clarify the mechanistic regulation of this interaction and how it integrates with known signaling cascades to modulate synaptic vesicle recycling and neurotransmitter release efficiency.

An important consideration when using NbLumSyt1 is that its signal should not be interpreted as “exclusively reporting *bona fide* SVs”, but rather as part of Syt1-positive membranes that are likely to be engaged in exo-endocytic and trafficking pathways. As previously mentioned, Syt1 is known not only to recycle SVs, but also to populate the plasma membrane in surface-retained pools, endosomal intermediates and post-synaptic pools. Depending on the experimental conditions, this may contribute to non-vesicular labelling. This is particularly relevant for approaches such as APEX2, where the labelling radius can amplify signals from adjacent compartments. Furthermore, overexpression conditions, developmental differences or altered trafficking dynamics may increase off-target nanobody accumulation. For these reasons, we recommend to incorporate in these experiments context-specific controls. These should ideally include validation in Syt1 KO backgrounds when possible, the use of non-binding nanobody controls (such as the Nb-ALFA-APEX2 in this study), activity-dependent modulation paradigms and benchmarking against other synaptic vesicle markers. Importantly, each application of NbLumSyt1 requires context-dependent validation. Together, these measures facilitate the interpretation of results and reduce false-positive detection, ensuring that NbLumSyt1 is applied with appropriate caution across different experimental modalities.

Single-molecule and nanoscale imaging using NbLumSyt1 tools further highlights their versatility. The distinct Syt1 populations with different mobility and clustering properties identified in this work align well with previous findings [10, 37, 38] and offer new insights into the organization and dynamics of SV proteins. Moreover, our study suggested that the overexpression of Syt1-pHluorin can affect single-molecule mobility and endocytosis. These overexpression-related artifacts are circumvented by using NbLumSyt1, which selectively binds the endogenous protein. These single-molecule approaches complement advanced imaging techniques, including MoNaLISA/RESOLFT microscopy [39, 46, 47], which allow live imaging nanoscale description together with other technologies recently developed, such as MINFLUX nanoscopy and SUM-PAINT, for extensive multiplexing of synaptic targets [40, 48]. By offering a nanobody-based methodology compatible with single-molecule imaging, this study extends the methodology repertoire when studying synaptic nanostructure and function at unprecedented resolution.

NbLumSyt1 is of particular translational interest. This demonstration of effective labeling within human iPSC-derived neurons ensures its applicability within human systems, opening the door for investigating SV recycling in more relevant synaptic neurodevelopmental and neurodegenerative disease models. Given the central role that SV dynamics play in both synaptic function and dysfunction [49], this could be a very valuable tool in the study of diseases such as Alzheimer’s disease, Parkinson’s disease, and autism spectrum disorders, where disturbances in SV recycling have been implicated. Importantly, NbLumSyt1 is further compatible with a wide range of experimental platforms, including live imaging, proteomics, and nanoscale analyses, thereby further increasing its versatility for basic and translational applications. The various approaches described in the NbLumSyt1 toolkit open the way to investigating neurodevelopmental processes, including synaptic maturation and pruning.

Overall, while NbLumSyt1 offers practical advantages over existing probes, the primary strength of this approach lies in its ability to enable multimodal, investigation of Syt1 biology across spatial scales and experimental platforms. For example, real-time imaging of SV recycling in developing neurons may reveal mechanisms underlying synaptic refinement during critical periods of brain development [50]. Such studies assume particular significance considering the emerging links between disrupted synaptic development and conditions such as schizophrenia and intellectual disabilities [51, 52]. Monitoring SV dynamics in neurodegenerative diseases, especially in the early asymptomatic phases [53], may provide insights into early synaptic dysfunction, which could identify biomarkers or therapeutic targets for conditions such as frontotemporal dementia and Huntington’s disease.

Future applications for NbLumSyt1 might include combining this tool with optogenetic or chemogenetic approaches [54] to manipulate and monitor the dynamics of SVs in real time. The adaptation of NbLumSyt1 will thus provide insights into synaptic function within intact neural circuits. Furthermore, the use of NbLumSyt1 in combination with advanced computational modeling will allow for quantitative analyses of SV recycling kinetics and their regulation under various physiological and pathological conditions. Beyond neuroscience, the experimental workflow that we have developed can inform broader uses, such as drug screening against synaptic proteins or studies of mechanisms in vesicle trafficking in other cellular contexts. In conclusion, NbLumSyt1 has significant transformative potential for the study of SV recycling because it presents a versatile, powerful platform for elucidating the molecular and functional basis of synaptic transmission. Its high specificity, noninvasiveness, and good adaptability make it stand out as a critical new tool for further advancements in the investigation of synaptic physiology and disease alterations.

## Online Methods

### Discovery of anti-luminal Syt1 nanobodies

Immunization and initial screenings were performed in a fee-for-service manner by NanoTag Biotechnologies GmbH (Göttingen, Germany). The intraluminal sequence of Syt1 from *Rattus norvegicus* was expressed and purified from *E. coli*. Two alpacas received six injections of 500 µg of total protein once a week. Two weeks after the last immunization, a final boost injection of 500 µg of antigen was used, and blood was drawn on days 3 and 5 postboost. PBMCs were isolated through a Ficoll gradient. Total RNA was extracted via a Qiagen RNA extraction kit. Using reverse transcription by SuperScript IV, total RNA extracted from PBMCs was used to generate cDNA. The final PCR product was verified on 1.5% agarose and then cloned and inserted into a phagemid via Gibson assembly. The resulting phagemid was electroporated into the bacteria TG1 for the generation of libraries. The transformed bacteria were subsequently grown in 2YT media supplemented with appropriate antibiotics, after which the resulting library was aliquoted and stored at − 80 °C. The library was panned for three rounds against the purified, biotinylated Syt1 antigen. M13KO7 helper phages infect the library to display nanobodies on phages. Phage precipitation was performed via PEG-8000, and purified phages were filtered after an overnight culture was performed. Dynabeads MyOne Streptavidin C1 captured the biotinylated antigen. Elution of the phage-bound antigen was used in subsequent rounds of selection. Finally, single clones were first characterized by ELISA using the antigen immobilized on a 96-well plate. Positive clones were sequenced, and candidates were produced for initial validation.

### Nanobody expression and purification

The selected nanobody clones expressed in NEBshuffle Express T7 *E. coli* (C3029) were transformed and grown overnight. A single colony was picked and grown in TB medium supplemented with 50 µg/mL kanamycin until the optical density at 600 nm (BioPhotometer 6131 Spectrometer, Eppendorf AG, Hamburg, Germany) reached 0.8. Expression was then induced with 0.3 mM isopropyl-β-D-1-thiogalactopyranoside (IPTG), and the cells were incubated for 14–16 h at 25 °C and 120 g in a shaker (Infors HT, Switzerland).

The nanobodies were purified via nickel binding affinity chromatography via a protocol adapted from [20]. The bacterial cells were subsequently centrifuged for 25 min at 4,000 × g and 4 °C (rotor: H-12000, Thermo Fisher Scientific, MA, USA). The pellet (15 g wet weight) was resuspended in 50 mL of resuspension buffer containing protease inhibitors and homogenized with a glass douncer three times manually. The cell resuspension was processed twice with the microfluidizer at 18,000 psi (M110- Microfluidizer, Microfluidics, MA, USA) and centrifuged at 40 K g at 4 °C for 30 min (rotor: Ti45, Beckman, California, USA). Nickel-NTA resin beads (HisPurTM Ni-NTA Resin, #88222; Thermo Fisher Scientific) were equilibrated with resuspension buffer by centrifuging for one minute at 800 × g on a tabletop centrifuge at 4 °C. This process was repeated three times to equilibrate the beads well. The cell supernatant was collected after centrifugation and rotated with equilibrated beads at 4 °C for 2–3 h. The supernatant bead suspension was then run over a 50 mL gravity flow column (Econo-column^®^ Bio-Rad Laboratories, Hercules, CA), and the flowthrough (FT) was collected. The beads were washed three times with 50 mL of wash buffer and once with cleavage buffer. After excessive washing, the beads were transferred to 10 mL of cleavage buffer in a 15 mL Falcon tube. One micromolar of Ulp1 (the yeast homolog of SUMO protease, purified in Dr. Alex Stein’s lab, MPI-BPC) was added, and the mixture was incubated for another hour at 4 °C with constant rotation. The beads were poured back into the column, and the flowthrough was collected. The samples were washed once again with 10 mL of cleavage buffer, the remaining eluted protein was collected, and 5 mM DTT was added. The nanobodies were further concentrated to 5 mL via a 3 kDa MWCO VivaSpin concentrator (Sartorius Stedim Biotech, Göttingen, Germany). The antibodies were additionally purified via ion-exchange chromatography on a MonoQ 10/100 GL column (GE Healthcare Life Sciences, Pittsburgh, PA) via an ÄKTA system (GE Healthcare Life Sciences, Pittsburgh, PA). ÄKTA buffers A and B were filtered (0.2 μm membrane filter, Sartorius Stedim Biotech, Göttingen, Germany) and degassed. The column was equilibrated with ÄKTA buffer A, and a protein sample was injected into the column. Elution was performed with a bdSUMO protease on a column [55], and the purity of the eluted proteins was assessed via SDS‒PAGE.

### Animals and cell culture

In most cases, neurons were cultured as described previously with minor modifications [56]. Briefly, postnatal day zero (P0) Wistar pups were sacrificed by decapitation, brains were extracted, and hippocampi were dissected and placed in an enzyme mixture containing papain for 30 min. Hippocampi were then transferred to inactivation medium. The digested tissue pieces were triturated via fire-polished glass pipettes, first with a larger hole and then with a fresh fire-polished pipette with a smaller hole, until there was a smooth, homogenous solution. The cells were passed through a 0.22 μm cell strainer, after which they were centrifuged at 5000 × g for 5 min. The supernatant was removed carefully, and the cell pellet was suspended in serum media. The cells were manually counted via a hemocytometer (Neubauer, Paul Marienfeld GmbH, Germany). A total of 20,000 cells/cm3 were added to the wells of a 12-well plate containing poly D-lysine (PDL)-coated coverslips containing neuron culture medium. The cells were grown at 37 °C in a 5% CO_2_ incubator and used for imaging between 14 and 18 DIV. For electron microscopy experiments, neuronal monolayer cultures from P0 C57/BL6J mice were cultured onto astrocyte feeder layers as previously described [57, 58]. Briefly, the astrocytes were grown on carbon- and PDL-coated 6 mm sapphire disks and glued onto glass coverslips using Matrigel at a density of 50,000/well in 12-well plates. Astrocytes were grown in DMEM-GlutaMax supplemented with 10% FBS and 0.2% penicillin‒streptomycin for six days before 80 µM FUDR was added overnight, after which the medium was replaced with Neurobasal‒A supplemented with 2% B27, 1% GlutaMax and 0.2% penicillin‒streptomycin (NB‒full medium, Invitrogen) an hour before the hippocampal neurons were plated. Hippocampi were prepared in ice-cold HBSS (CaCl_2_- and MgCl_2_-) and incubated in papain at 37 °C for 30–60 min on a shaker; the mixture was triturated, and 100,000 HBSS/well were plated onto the astrocyte feeder layer. Cultured neurons were vitrified by high-pressure freezing at DIV 14.

To compare the properties of NbLumSyt1-pHluorin with those of Syt1-pHluorin, primary dissociated hippocampal cultures were prepared from mouse embryos (C57BL/6J, E17.5). The mouse hippocampi were digested with papain (10 U/ml) in Dulbecco’s phosphate-buffered saline (PBS), rinsed with minimal essential medium containing 10% fetal bovine serum (FBS), and then mechanically triturated to obtain a single-cell suspension. The cells were plated on 25 mm diameter coverslips coated with poly-D-lysine and laminin. Cultures were maintained in neurobasal medium supplemented with 2% B-27, 0.5 mM L-glutamine, and 1% (v/v) penicillin‒streptomycin, with 1 mM cytosine β-D-arabinofuranoside added at 3 days in vitro (DIV) to inhibit glial proliferation. Neurons were transfected at DIV7 via Lipofectamine 2000 (Thermo Fisher Scientific, 11668-019) according to the manufacturer’s instructions. Imaging of these neurons was performed between DIV14 and DIV16.

### BCA protein estimation

A Pierce™ BCA protein assay kit (#23225, Thermo Fisher Scientific, MA, USA) was used to estimate the concentration of proteins according to the manufacturer’s instructions in a 96-well flat-bottomed plate (Greiner GmbH, Germany). Protein standard buffers (#23208, Thermo Fisher Scientific, MA, USA) were used to plot a BSA standard curve. The absorbance at 650 nm was measured in a plate reader (Tecan Genios Pro, Männedorf, Switzerland) according to the manufacturer’s manual (BCA Protein Assay, 2020) modified from [59].

### SDS Schägger Gels

Protein samples were analyzed via SDS‒PAGE on Schägger gels [60]. Protein samples (10 µg) were mixed with loading buffer and run on a 10% resolving gel and a 4% stacking gel. The gels were run at 60 V until the samples entered the resolving gel, after which they were run at 120 V (Bio-Rad Laboratories, Hercules, CA). The PageRulerTM prestained protein ladder 10–180 kDa (#26617, Thermo Fisher Scientific, MA, USA) was used as a molecular marker. The gels were stained by being incubated with Coomassie blue and boiled in a microwave for one minute, followed by shaking for 10 min at RT. They were then incubated in destaining buffer 1 and again boiled for one minute, followed by shaking for 30 min. Finally, they were destained in destaining buffer 2 overnight and then scanned on a scanner (HP, California, US).

### Western blotting

The gels were transferred to a nitrocellulose membrane after being run on Schägger gels according to the methods of [61] with the following modifications. A TransBlot Turbo Transfer machine (Bio-Rad Laboratories, Hercules, CA) was used to perform the transfer within 7 min according to the manufacturer’s instructions. The membrane was blocked in 5% milk in TBST/PBST. Furthermore, the membranes were incubated with the required primary antibody prepared in 5% milk in TBS/TBST/PBST (depending on Ab stability, TBS or PBS (with or without Tween) was used) at 4 °C overnight or for 1–2 h at RT. The membranes were then washed with TBST/PBST and incubated with secondary infrared (IR) dye-labeled antibodies prepared in TBS/PBS for an hour at RT. After three washing steps of 15 min each, the fluorescent protein bands were visualized via an Odyssey CLx IR Imaging System (LI-COR Biosciences, GmbH, Germany).

### Immunoprecipitation of Syt1 from WT and KO Syt1 mouse brains using nanobodies against the luminal domain of Syt1

Syt1 WT and KO mouse brains were provided by the lab of Prof. Volker Hauke, Berlin, Germany. The protocol for Syt1 immunoprecipitation (IP) was adapted from Abcam (Cambridge, UK) (Abcam, 2010) and modified. GFP-tagged nanobody (Nb) was preloaded on the beads, and 20 µL of bead mixture (GFP Selector, # N0130, NanoTag Biotechnologies, Germany)/sample was preincubated with 5 µL of GFP Nb (100 µM, NanoTag, cat. N0305-Biotin, RRID: AB_3075908) in 75 µL of lysis buffer. The ‘No Nb’ control was also prepared in the same way. The bead slurry with Nb was constantly agitated for 30 min at 4 °C on an orbital shaker in a cold room. The mixture was then centrifuged for one minute at 1000 × g (ThermoScientificTM HeraeusTM), and the supernatant was carefully removed. One milliliter of lysis buffer was added, and the mixture was rotated for 5 min at 4 °C. The beads were ready to use after being centrifuged once more for one minute at 1000 × g. Lysates from the tissue–frozen brains were thawed, and 300 µL of lysis buffer per brain was added to an Eppendorf tube (Sarstedt AG & Co. KG, Germany). The brains were homogenized via a small Teflon homogenizer with 3–4 strokes up and down manually. Debris and nuclei were pelleted by centrifugation at 1000 × g (ThermoScientificTM HeraeusTM) for 5 min, and the resulting supernatant was used for protein extraction. The supernatant was constantly agitated for 30 min at 4 °C on an orbital shaker, followed by centrifugation for 10 min at 12,000 × g in a microcentrifuge (ThermoScientificTM HeraeusTM). The tubes were removed and kept on ice, and the supernatant was transferred to fresh, cold, low-binding Eppendorf tubes. The protein concentration was estimated via a BCA protein estimation kit as described previously. Preclearing the lysate helps reduce nonspecific binding and reduces the background. One hundred microliters of lysate were mixed with naked (not preloaded Nb) bead slurry for 30 min on an orbital shaker at 4 °C. The beads were sedimented by spinning at 1000 × g for one minute, and the supernatant was used for the IP. Each sample (50 µL of lysate and 20 µL of slurry) was incubated at 4 °C for an hour on an orbital shaker. The beads were then sedimented by centrifugation at 1000 × g for one minute and resuspended in 1 mL of lysis buffer in a fresh Eppendorf tube. The mixture was again centrifuged for one minute at 1000 × g, and the supernatant was discarded. This washing step was repeated one more time. The beads were finally resuspended in 20 µL of SDS buffer and heated at 95 °C for 5 min. The samples were loaded on an SDS gel, and western blotting was performed using Syt1 cytoplasmic (SynapticSystem, cat. #105 011, AB_887832) and GAPDH (ThermoScientificTM, cat. MA5–15738, RRID: AB_10977387) antibodies.

### Fluorometry for mOrange2/pHlourin pH calibration

Fluorometry was performed on a Fluorolog^®^-3 instrument (Horiba Scientific, Kyōto, Japan) with 250 µL glass cuvettes (Starna GmbH, Germany) to minimize the volume of the precious fluorophores and proteins. In vitro pH calibration of the pHlourin/mOrange2 fusion proteins was performed as previously described [27] with slight modifications. MES buffer was used for pH values of 5, 5.6, and 6.2, and HEPES buffer was used for pH values above 6.8. The concentration of 0.8 µM of the two proteins was enough to give a substantial number of counts (> 100 K counts) on the Fluorolog. The protein was added to 250 µL of each of the pH-clamped buffers in Eppendorf tubes and vortexed briefly. The mixture was placed in a cuvette, and the emission spectra were recorded. The data were analyzed via Graph Pad Prism.

### Nb binding with HaloTag fluorescent ligands

HaloTag fused with NbLumSyt1 (NbLumSyt1-HALO) was produced and purified via NanoTag Biotechnologies. The NbLumSyt1-HALO and HaloTag fluorescent ligands were mixed in equimolar ratios at a final concentration of 10 µM for 15 min at RT. Complex formation was confirmed via fluorescence imaging. HALO-Tag ligands were purchased from Promega.

### Labeling of cultured neurons with Nb/Ab by spontaneous endocytosis

Neurons were labeled as described previously [62], although with the following modifications. Two hundred microliters of media from an 18 mm diameter coverslip (Karl Hecht Assistant, Germany) containing cultured hippocampal neurons were placed in an Eppendorf tube and mixed with the required labeled antibody (Ab) or a nanobody (Nb) (5 µM final concentration). The mixture was subsequently centrifuged at 13,000 × g for 10 min at 37 °C to remove the dye aggregates. The coverslip was incubated in the media for 10 min and placed in a 37 °C/5% CO_2_ incubator to label the recycling pool of SVs. The neurons were then washed quickly 3 times with warm Tyrode’s buffer (124 mM NaCl, 5 mM KCl, 30 mM glucose, 25 mM HEPES, 2 mM CaCl2, and 1 mM MgCl2, pH 7.4). Experiments were performed on neurons from 14 to 18 DIV. Imaging was performed instantly in warm Tyrode’s buffer.

### Immunostaining

For immunostaining (Fig. 1), the cells were fixed with 4% (w/v) paraformaldehyde (PFA; P6148, Sigma‒Aldrich) in 4% sucrose (0.12 M) in PBS for 20 min at RT, followed by washing three times with 1× PBS. Excess PFA was quenched with 100 mM NH_4_Cl for 20 min, and the samples were then washed three times with PBS. Unspecific binding was blocked by incubating the coverslip in 2.5% BSA and 0.1% Triton X-100 in PBS for 15 min. The cells were then incubated in a humidified chamber at RT for 1 h in primary antibodies diluted in 2.5% BSA and 0.1% Triton in PBS. The cells were subsequently washed twice with 2.5% BSA and 0.1% Triton X-100 in PBS and once with 2.5% BSA in PBS. Finally, the coverslips were incubated for 1 h in a humidified chamber at RT with secondary Abs (1:200, unconjugated from Jackson ImmunoResearch and conjugated in house as previously described in (Coons et al. [63]), donkey anti-mouse IgG: cat# 715-005-151, RRID: AB_2340759; donkey anti-rabbit IgG: cat# 711-005-152, RRID: AB_2340585; donkey anti-guinea pig IgG: cat# 706-005-148, RRID: AB_2340443) in 2.5% BSA in PBS. After the coverslips were washed three times with 2.5% BSA in PBS, they were washed once with high-salt PBS (400 mM NaCl), washed again with PBS, and finally mounted on slides with mounting medium. Coverslips were incubated at RT for a few hours, incubated at 4 °C overnight and imaged the following day. The primary antibodies used were anti-Syt1 clone 604.2 (105102, Synaptic Systems) and anti-VGAT (Synaptic Systems, cat. #131103, RRID: AB_887870).

### General epifluorescence and confocal microscopy imaging

Images were acquired on a Nikon Ti 2E (Nikon Corporation, Chiyoda, Tokyo, Japan) inverted epifluorescence microscope equipped with a 60X oil objective and a Photometrics 95B camera. A dark Okolab (Ottaviano, Italy) cage incubator system was used to maintain 37 °C and 5% CO2 (OKO Air Pump and CO2 controller). GFP/pHlourin was imaged in the Alexa488 channel (EX 472/30, DM 495, BA 520/35), whereas the mOrange2 probe was imaged in the Cy3 channel (EX 531/40, DM 562 BA 593/40). Alexa647 was imaged in the Cy5 channel (EX 628/40, DM 660, BA 692/40). Imaging of the fixed samples was also performed on a Zeiss LSM710 laser scanning confocal microscope with a scanning format of 1,024 × 1,024 pixels using a 63x oil-immersion objective with a 1.3 numerical aperture (NA) at equal acquisition settings within each immunostaining.

### Stimulation assays for testing the effects of activity on the localization of SVs

Prior to electrical stimulation, neurons were rapidly washed with buffer containing 50 µM APV and 10 µM CNQX to avoid recurrent firing and then exposed to the same buffer along with the premixed NbLumSyt1-HALO-JF646. Following the addition of NbLumSyt1-HALO-JF646, neurons were immediately stimulated at 20 Hz for 2 s. In the control condition, neurons were stimulated only in the presence of the unconjugated HaloTag JF646. After stimulation, neurons were allowed to recover for 2 min in the same buffer, washed twice with Tyrode’s buffer and fixed. After fixation and quenching, the neurons were stained with an anti-Bassoon antibody and a VGLUT1-STAR580 nanobody. The surface epitopes of Syt1 were saturated by incubating the neurons in divalent-free buffer (Tyrode buffer without Mg^2+^ or Ca^2+^) with HaloTag-free NbLumSyt1 for 5 min at 37 °C. Until fixation, the neurons were kept at 37 °C on a heating metal plate. Before stimulation, 15 µl of NbLumSyt1-HALO was premixed with 3 µl of Janelia Fluor 646 HaloTag ligand (100 pmol of JF646 stock at 33 µM, Promega GA112A) for 15 min at RT and diluted to 300 µl final volume in Tyrode (to reach 4.85 µM final concentration of NbLumSyt1-HALO-JF646). Neurons were quickly washed with drug buffer containing 50 µM APV and 10 µM CNQX to avoid recurrent activation. The neurons were stimulated at 20 Hz for 30 s. After stimulation, the neurons were allowed to recover for 2 min in the same buffer, washed two times with Tyrode’s solution and fixed with 4% PFA for 30 min at RT. The following steps were performed at RT. Neurons were quenched with 100 mM NH_4_Cl solution, quickly washed with PBS and blocked with blocking solution containing 2% bovine serum albumin (BSA, PanReacAppliChem) and 0.1% Triton X-100 (Sigma Aldrich, cat. 102604816) for 15 min. Afterwards, the neurons were incubated in blocking solution containing mouse anti-bassoon antibodies (1:200 dilution, Enzo ADI-VAM-PS003-F, RRID: AB_1659574) for 1 h. Then, the neurons were washed three times in PBS for 5 min. Afterwards, the neurons were incubated in blocking solution containing donkey anti-mouse Alexa Fluor 488 minimal cross-reactivity antibodies (AF488, 1:500 dilution, Jackson ImmunoResearch 715-545-151) and vGlut1-STAR580 nanobodies (1:500 dilution, FluoTag-X2 N1602-Ab580-L, NanoTag Biotechnologies, RRID: AB_3076007 (custom-labeling order)) for 1 h. Then, the neurons were washed three times in PBS for 5 min and mounted. The coverslips were dipped in ddH_2_O to remove excess salt, and the side of the coverslip was quickly dried on a Kim wipe to remove excess liquid. Immediately after, the coverslips were mounted on a microscope slide using 10 µl of Prolong Glass Antifade mounting media (Thermo Fisher, cat. P36980), left to harden overnight at RT and kept at 4 °C until imaging.

### Immunolabeling assay for Cntfr and the luminal domain of Syt1

To label Cntfr, 15 DIV primary hippocampal neurons were incubated with rabbit anti-CNTFRα (extracellular) antibodies (1:50 dilution, Alomone ACR-051, RRID: AB-2340925) in conditioned media at 37 °C for 40 min. Neurons were washed in cold Tyrode’s buffer and fixed with 4% PFA for 30 min at room temperature (RT). The following steps were performed at RT. Neurons were quenched with 100 mM NH_4_Cl solution, quickly washed with PBS and blocked with blocking solution containing 2% bovine serum albumin (BSA; Merck 1120180500) and 0.1% Triton X-100 (TX-100; Merck 1122980101) for 15 min. Afterwards, the neurons were incubated in blocking solution containing mouse anti-Syt1 luminal (1:200 dilution; Synaptic Systems, cat. #105201 604.1 clone; RRID: AB_2617068) antibodies for 1 h. Then, the neurons were washed three times in PBS for 5 min. Afterwards, the neurons were incubated in blocking solution containing anti-rabbit STAR635P (1:170 dilution, in-house conjugated rabbit Jackson ImmunoResearch 711-005-152 and STAR635P NHS Abberior 07679) and anti-mouse STAR580 (1:200 dilution, in-house conjugated mouse Jackson ImmunoResearch 715-005-151 and STAR580 NHS ester Abberior 38377) for 1 h. Then, the neurons were washed three times in PBS for 5 min and mounted as described above.

Confocal and STED images were acquired via an Abberior microscope setup (Abberior Instruments GmbH), including an Olympus IX83 microscope body and Imspector software (version 16.3.15521–w2209). Using a UPLSAPO100XO objective (1.4 NA), 10 single z-plane images of 20 μm × 20 μm image size and 20 nm × 20 nm pixel size were collected per condition. The STAR580 and STAR635P fluorophores were excited via 561 nm and 640 nm laser lines and depleted via the 775 nm laser line, and the emission was detected in the 605–625 nm and 650–720 nm ranges, respectively, in the STED mode.

Image analysis was performed via an in-house written ImageJ/Fiji (citation) macro. Briefly, binary masks of Syt1- and Cntfr-positive regions were obtained by applying a 1 sigma radius Gaussian blur filter and setting a user-defined threshold. These objects were filtered by area (0.04–2 µm^2^ for Syt1 and 0.01–0.8 µm^2^ for Cntfr) and counted for every image. An overlap between Syt1 and Cntfr was manually identified by selecting a fixed size circle ROI around the overlap. These regions were counted and expressed as percentages over total Syt1.

### Proximity ligation assay (PLA) for Cntfr and Syt1

Labeling of Cntfr was also performed as described above, including mouse anti-Syt1 luminal antibodies (1:150 dilution, Synaptic Systems, cat. #105 201, 604.1 clone, RRID: AB_2617068) in conditioned media at 37 °C for 40 min. In the control conditions, neurons were incubated with rabbit anti-ribeye (1:62.5 dilution, Synaptic Systems, cat. #192 003, RRID: AB_2261205) antibodies instead of Cntfr antibodies.

The neurons were washed, fixed, quenched and permeabilized as described above. Then, the neurons were subjected to a proximity ligation assay via a Duolink™ In Situ Orange Starterkit Mouse/Rabbit (DUO92102-1KT, Merck) according to the Duolink^®^ PLA Fluorescence Protocol. After the final washes, the neurons were incubated in blocking solution containing guinea pig anti-synaptophysin1 (1:500 dilution, Synaptic Systems, cat. #101 004, RRID: AB_1210382, polyclonal antiserum) antibodies for 1 h. The neurons were subsequently washed three times in PBS for 5 min. The neurons were subsequently incubated in blocking solution containing anti-guinea pig AF488 (1:200 dilution, minimal cross-reactivity Jackson ImmunoResearch cat no: 706-545-148) antibodies for 1 h. Then, the neurons were washed three times in PBS for 5 min and mounted as described above.

Epifluorescence images were acquired via an Olympus microscope setup including an Olympus IX71 microscope body and OLYMPUS cellSens Dimension (version 2.3) software. Using a UPlanSApo 60× objective (1.35 NA), 10 single z-plane images of 147.92 μm × 111.59 μm image size and 0.1075 μm × 0.1075 μm pixel size were collected per condition. The fluorophores were excited via a Lumencor SOLA SE II lamp. The emissions of the Duolink Orange fluorophores were collected for a 200 ms exposure time in a range with an emission peak of 580 nm, and the emissions of the AF488 fluorophores were collected for a 100 ms exposure time in a range with an emission peak of 518 nm.

Image analysis was performed via an in-house written ImageJ/Fiji macro. Briefly, binary masks of Synaptophysin- and PLA-positive regions were obtained by subtracting the background (rolling ball radius of 10 pixels), applying a 1 sigma radius Gaussian blur filter on the Synaptophysin signal and setting a user-defined threshold. These objects were filtered by area (0.05–2 µm^2^ for Synaptophysin and larger than 0.03 µm^2^ for PLA) and counted for every image. The overlap between Synaptophysin and the PLA was obtained by performing an “AND” operation between two masks, and the colocalizing regions were counted. The number of such regions was divided by the total number of Synaptophysin regions and normalized by the average of the control condition. The results were plotted as boxplots showing the means and 5th and 95th percentiles of the dataset. Unpaired t tests were performed via GraphPad Prism v9.4.0.673 for Windows (GraphPad Software, San Diego, California, USA).

### Pharmacological alteration of Cntfr and Syt1 contacts

At 14 days in vitro (DIV), the neurons were incubated either in conditioned medium only (control) or in conditioned medium supplemented with 8 nM ciliary neurotrophic factor (CNTF, C-245 Alomone Labs) for 2–24 h or with 3 µM tetrodotoxin (TTX, 1069 Tocris) for 2 h. As a proxy for neuronal activity, neurons were exposed to an anti-Syt1 luminal primary antibody directly labeled with ATTO647N (1:200, 105 311AT1 clone 604.2 Synaptic Systems) in conditioned medium for 30 min at 37 °C. Later, the cells were washed, fixed, quenched, stained for Synaptophysin 1 and mounted as described above.

Imaging was performed using a Plan Apo λ 60× oil (NA 1.4) objective and an Andor DU-897 X-8536 camera with an inverted Nikon Ti microscope (Nikon Corporation, Chiyoda, Tokyo, Japan). GFP and CY5 filters were used to excite the Alexa Fluor 488 and ATTO647N fluorophores, respectively. Images of 135.21 × 135.21 μm size with a pixel size of 26.4 × 26.4 nm were collected via NIS Elements software.

All the images were analyzed via a semiautomated Fiji macro. Synaptophysin-positive regions were identified, and a common threshold was set. For general intensity analysis, Syt1 fluorescence intensity (a.u.) was measured in the whole synaptophysin-positive region. For bouton-specific analysis, the Syt1 fluorescence intensity (a.u.) and area (µm^2^) were measured in every synaptophysin-positive bouton. A 2 µm^2^ maximum area filter was applied to exclude boutons that might have been merged during thresholding. Boutons were divided into three groups: small (< 0.4 µm^2^), medium (0.4–0.9 µm^2^) and large (> 0.9 µm^2^). An average of the mean intensity was calculated for every group in every image (*n* = 30 images), in every condition (*N* = 3 coverslips), normalized to the median of the control and expressed as a percentage. One-way ANOVA followed by a post hoc Bonferroni multiple comparisons test was performed via GraphPad Prism v9.4.0.673 for Windows (GraphPad Software, San Diego, California, USA).

### Electrical stimulation of cultured neurons

A perfusion-type electrical stimulation magnetic chamber (EC B18, Live Cell Instrument, Gyeonggi-do, The Republic of Korea) was used to achieve field stimulation at the indicated frequencies and pulses with an A310 Accupulser Stimulator (Precision Instruments, Sarasota, FL, USA). Stimulation was performed in the presence of CNQX and APV to reduce recurrent activity from excitatory synapses. One image every 2 s was captured for a total of 3.5 min. After the first 30 s, electrical stimulation was applied, and 3 min later, 50 mM NH_4_Cl was applied. For the bafilomycin A1 control experiment, 250 nM bafilomycin A1 was included immediately before image acquisition in Tyrode’s buffer. The exposure times and light intensities were adjusted to minimize photobleaching. The ‘perfect focus system’ feature was used before the start of the experiment to avoid any focal drift caused by stimulation, the addition of NH_4_C, or a change in pH/Cl- clamped buffers.

### Electrophysiology

Whole-cell recordings of pyramidal neurons were obtained via Axopatch 200B and Clampex 8.0 software (Molecular Devices), filtering at 1 kHz and sampling at 5 kHz with voltage clamping at -70 mV. Only experiments with access resistance values of 5–20 MΩ were considered for analysis. All recordings were performed at room temperature. The composition of the internal pipette mixture was 115 mM CsMeSO_3_, 20 mM tetraethylammonium chloride, 10 mM CsCl, 10 mM HEPES, 5 mM NaCl, 0.6 mM EGTA, 4 mM Mg-ATP, 0.3 mM Na2GTP and 10 mM QX-314 (lidocaine N-ethyl bromide). The final solution was adjusted to a pH of 7.3 and an osmolarity of 305–310 mOsM. The final resistance of the electrode tips used was ~ 2–5 MΩ. To elicit evoked responses, electrical stimulation was delivered via a parallel bipolar electrode (FHC) and a constant current unit (WPI A385) coupled with a Master-8 controller (A.M.P.I.). The pulse duration was 0.1 ms, and the intensity was 35 mA (the same stimulation equipment and parameters were used for the live fluorescence imaging and electrophysiology experiments in Fig. 3a-g). The extracellular solution was a modified Tyrode’s solution containing 150 mM NaCl, 4 mM KCl, 10 mM glucose, 10 mM HEPES and 2 mM MgCl2, adjusted to pH 7.4 and 315–320 mOsM. The agonists against the ionotropic glutamate receptors 6-cyano-7-nitroquinoxaline-2,3-dione (CNQX, 10 µM) and aminophosphonopentanoic acid (AP-5, 50 µM) were used to isolate inhibitory postsynaptic currents (IPSCs). To isolate excitatory (AMPA-mediated) postsynaptic currents (EPSCs), the GABA-A receptor inhibitors picrotoxin (PTX, 50 µM) and AP-5 were added to the bath solution. Spontaneous neurotransmission was recorded after the addition of 1 µM TTX. Evoked recordings were analyzed via Clapfit (Molecular Devices), and miniature events were analyzed via MiniAnalysis (Synaptosoft).

### Nanobody uptake and post hoc immunostaining

For NbLumSyt1 live-cell uptake at DIV 14, the corresponding coverslip was incubated in 200 µL of CM diluted 1:200 with GFP-tagged nanobody for 30 min at 37 °C and 5% CO2. After incubation, the coverslip was transferred to the original medium for 15 min, and for labeling, the neurons were washed three times with 1x PBS and fixed with 4% PFA/4% sucrose in PBS for 15 min at room temperature (RT). Each coverslip was washed three times with PBS, blocked with blocking solution (1x PBS, 10% normal goat serum (NGS) and 0.3% Triton X-100) for 30 min at RT and incubated with primary antibodies (anti-GFP (rabbit polyclonal, used at 1:1000 in IF, Abcam, Cat# ab6556, RRID: AB_305564) and anti-vGLUT1 (guinea pig polyclonal, used at 1:500 in IF, Synaptic Systems, Cat# 135 304, RRID: AB_887878)) diluted in blocking solution for 2 h at RT. Before and after incubation with the corresponding secondary antibodies (goat anti-rabbit IgG Alexa Fluor 488 (polyclonal, used at 1:400, Thermo Fisher Scientific, Cat# A-11008, RRID: AB_143165) and goat anti-guinea pig IgG Alexa Fluor 568 (polyclonal, used at 1:400, Thermo Fisher Scientific, Cat# A-11075, RRID: AB_141954)) for 45 min, each coverslip was washed three times with PBS and finally mounted on glass slides in Immumount (Thermo Fisher Scientific).

### Automated solid-phase peptide synthesis

The µSPOT peptide arrays [64] were synthesized via a MultiPep RSi robot (CEM GmbH, Kamp-Lintford, Germany). As a substrate, we used acid-labile, amino-functionalized cellulose membrane discs containing 9-fluorenylmethyloxycarbonyl-β-alanine (Fmoc-β-Ala) linkers (with an average loading of 130 nmol/disc with spots of 4 mm in diameter) produced in house. Synthesis was initiated by Fmoc deprotection using 20% piperidine (pip) in dimethylformamide (DMF), followed by washing with DMF and ethanol (EtOH). Peptide chain elongation was achieved via a coupling solution consisting of preactivated amino acids (aa, 0.5 M) with ethyl 2-cyano-2 (hydroxyimino) acetate (oxyma, 1 M) and N, N’-diisopropylcarbodiimide (DIC, 1 M) in DMF (1:1:1:1, aa: oxyma: DIC). Coupling was carried out for 3 × 30 min, followed by capping (4% acetic anhydride in DMF) and washing with DMF and EtOH. Synthesis was finalized by deprotection with 20% pip in DMF (2 × 4 µL/disc for 10 min each), followed by washing with DMF and EtOH. The dried discs were transferred to 96 deep-well blocks and treated while shaken with side-chain deprotection solution consisting of 90% trifluoracetic acid (TFA), 2% dichloromethane (DCM), 5% H_2_O and 3% triisopropylsilane (TIPS) (150 µL/well) for 1.5 h at room temperature (rt). Afterward, the deprotection solution was removed, and the discs were solubilized overnight (ON) at RT while shaking. A solvation mixture containing 88.5% TFA, 4% trifluoromethanesulfonic acid (TFMSA), 5% H_2_O and 2.5% TIPS (250 µL/well) was used. The resulting peptide‒cellulose conjugates (PCCs) were precipitated with ice-cold ether (0.7 mL/well) and spun down at 2000 × g for 10 min at 4 °C, followed by two additional washes of the formed pellet with ice-cold ether. The resulting pellets were dissolved in DMSO (250 µL/well) to obtain the final stock solutions. PCC solutions were mixed 2:1 with saline–sodium citrate (SSC) buffer (150 mM NaCl, 15 mM trisodium citrate, pH 7.0) and transferred to a 384-well plate. For transfer of the PCC solutions to white-coated CelluSpot blank slides (76 × 26 mm, Intavis AG Peptide Services GmbH and Co. KG), a SlideSpotter (CEM GmbH) was used. After completion of the printing procedure, the slides were left to dry ON.

### Peptide microarray binding assay

Before use, the microarray slides were blocked for 60 min in 5% (w/v) skim milk powder (Carl Roth) in phosphate-buffered saline (PBS; 137 mM NaCl, 2.7 mM KCl, 10 mM Na_2_HPO_4_, and 1.8 mM KH_2_PO_4_, pH 7.4). After blocking, the slides were incubated for 30 min with 7.36 nM ALFA-NbLumSyt1 in blocking buffer and then washed three times with PBS. ALFA-NbLumSyt1 was detected with a secondary 1:50000 dilution of the nanobody anti-ALFA-HRP (NanoTag Biotechnologies, cat. N1505-HRP, RRID: AB_3075989). The nanobodies were applied in blocking buffer for 30 min, with three washes with PBS between the nanobodies and the application of the secondary nanobody. The chemiluminescent readout was obtained via SuperSignal West Femto maximum sensitive substrate (Thermo Scientific GmbH, Schwerte, Germany) with a c400 Azure imaging system (lowest sensitivity, 10 s exposure time). Binding intensities were quantified with FIJI [65] via the “microarray profile” plugin (OptiNav Inc., Bellevue, WA, USA). The raw grayscale intensities for each position were obtained for the left and right sides of the internal duplicate on each microarray slide, *n* = 4 arrays in total.

### Expression and purification of the nanobody-Syt1 luminal peptide complex

The DNA sequences encoding NbLumSyt1 and the 6x histidine-tagged Syt1 luminal peptide (MGEGKEDAFSKLKEKFMNELHKGHHHHHH) were subsequently cloned and inserted into the pETDuet-1 vector. The NbLumSyt1-Syt1 luminal peptide complex was expressed in *E. coli* BL21(DE3) via standard IPTG induction and purified via immobilized metal affinity chromatography (IMAC) followed by size exclusion chromatography (SEC) via a Superdex75 16/60 column (GE Healthcare) equilibrated with 20 mM HEPES pH 7.4, 150 mM NaCl, and 5% (v/v) glycerol. The purified complex was concentrated to 43 mg/ml for crystallization.

### Crystallization and structure of the nanobody-Syt1 luminal peptide complex

The NbLumSyt1-Luminal peptide complex (43 mg/mL) was crystallized at 21 °C via vapor diffusion in a 24-well sitting drop plate, where 1 µL of protein mixture was mixed with 1 µL of reservoir solution (1.6 M DL-malic acid). X-ray diffraction data were collected at station I04 of the Diamond Light Source (Oxford, UK) equipped with a Dectris Eiger2 XE 16 M detector. The data were processed and scaled with DIALS [66] and Aimless [67] within the CCP4 suite [68]. Molecular replacement was performed in Phaser [69] using the structure with the PDB code 6I2G as the search model [20]. Several cycles of model building and refinement were carried out via Coot [70] and Refmac5 [71]. The data collection and processing statistics are presented in Supplementary Table 2. Figures and analysis are based on chains.

A (nanobody) and I (peptide), which have the best electron density in the structure. The coordinates and structural factors for NbLumSyt1 were deposited in the PDB under the accession code 8B8I.

### Coupling of the NbALFA with nanogold particles

As described above, NbALFA was produced in SHuffle cells, bearing a free ectopic Cys on its C-terminus. NbALFA-Cys was reduced with 10 mM TCEP for 1 h. The excess TCEP was removed via a Nap5 (Cytiva) column, immediately mixed with an equimolar amount of monofunctionalized maleimide 3 nm gold particles (Nanopartz, Loveland, US) and left to react for two hours at room temperature. Nonconjugated gold was removed via a size exclusion column (Superdex 75 Increase 10/300 GL, Cytiva). NbALFA-Gold was kept at 4 °C.

### High-pressure freezing, freeze substitution, and ultramicrotomy

Sapphire discs with neurons were incubated in premixed NbLumSyt1-ALFA-tag Nb [5 µM]: NbALFA-Gold [3 µM] complex in conditioned medium or in conditioned medium containing only NbALFA-Gold [3 µM] for 45 min. The neurons then underwent washing steps in prewarmed (37 °C) conditioned medium before rapid cryofixation via an EM ICE high-pressure freezer (Leica) and storage in liquid nitrogen. Semiautomated freeze-substitution was performed via an AFS2 device (Leica) equipped with customized aluminum sapphire disc revolvers, and the samples were subsequently embedded in epoxy resin according to a previously published protocol [72]. One minor modification implemented to enhance the membrane contrast entailed the addition of 1% water [73] to the sequential freeze-substitution cocktails of 0.1% tannic acid and 2% osmium tetroxide in glass-distilled acetone. Upon sapphire disc removal from the polymerized samples, the block faces were trimmed with an EM TRIM high-speed milling device (Leica) in preparation for ultramicrotomy. A 35° Ultra Semi diamond knife (Diatome) mounted on a UC7 ultramicrotome (Leica) was used to cut alternating series of sections onto formvar-coated copper mesh grids at two different thicknesses: (i) 60-nm-thick sections were visually inspected to assess ultrastructural preservation and synapse density under an 80 kV LEO 912 transmission electron microscope (Zeiss), and (ii) 250-nm-thick sections were coated on both surfaces with protein A-conjugated 10-nm gold fiducial particles (Cell Microscopy Core Products, University Medical Center Utrecht, The Netherlands) in preparation for transmission electron tomography.

### Electron microscopy

Synapses were identified and selected for 3D ultrastructural analysis using tiled overviews acquired at 11000x magnification using a 200 kV Talos F200C G2 scanning/transmission electron microscope equipped with a 16 MP Ceta CMOS camera (Thermo Scientific) and MAPS v3.1 software (Thermo Scientific). Tilt series (± 60° tilt range; 1° increments) were acquired at 36,000 × (unbinned pixel size = 0.4 nm) or 57,000 × magnification (unbinned pixel size = 0.25 nm) from orthogonal axes via a Model 2040 dual-axis specimen holder (Fischione) and TEM Tomography v4 software (Thermos Scientific). Tomograms were generated from tilt series by using Gaussian filtering and a weighted back-projection algorithm implemented via the *Etomo* software IMOD software package [74]. The acquired 36000x magnification (*n* = 39 and *n* = 36 for the NbALFA-Gold and NbLumSyt1-ALFA-tag: NbALFA-Gold complexes, respectively) and 57000x magnification tomograms (*n* = 11 for the NbLumSyt1-ALFA-tag: NbALFA-Gold complexes) were analyzed via 3dmod software and additional programs within the IMOD software package [75]. For visualization purposes, tomograms were either binned to enhance membrane contrast or analyzed in an unbinned state to facilitate the discrimination of individual gold particles. SynapseNet [76] was exploited to annotate synaptic vesicles within tomographic subvolumes. Gold particles were detected via *findbeads3d* and clusters (≥ two gold particles) were identified using DBSCAN implementation of the Scipy library (eps = 10 nm, minPts = 2) [77]. It should be noted that our findbeads3d approach is prone to underestimate gold particle numbers in large clusters where the edges of individual particles are difficult to discriminate. Gold cluster volumes were quantified using *contourmod* and *imodinfo*. In this thresholded approach, gold cluster volumes likely represent underestimates given that stringent gray value thresholds were implemented to circumvent spurious detection of other high-contrast voxels of non-gold origin. Clusters were sorted on the basis of their location (intracellular, extracellular, presynaptic, postsynaptic, SVs, endosomes and multivesicular bodies). The active zones and clear-core vesicles were segmented via *3dmod*, and their diameters and closest approach distances were quantified via the *imodinfo* and *mtk* programs, respectively.

### Live imaging of synaptic vesicle exo- and endocytosis

For the initial experiments, fluorescence was recorded via a Nikon Eclipse TE2000-U microscope (Nikon) and an Andor iXon+ back-illuminated EMCCD camera (Model no. DU-897E-CSO-#BV). For illumination, we used a Lambda-DG4 illumination system (Sutter instruments) with a FITC emission filter. Images were acquired at 5 Hz. Solutions were perfused via an automatic, constant flux system (AutoMate Scientific). Circular regions of interest (ROIs) 2 μm in diameter were drawn around local fluorescence maxima (putative presynaptic boutons) and measured via Fiji (NIH). The fluorescence peaks synchronous with the stimulation were detected and analyzed via MATLAB. For additional experiments, live and actively recycled neurons were continuously incubated for 15–30 min or transfected with Syt1-pHluorin. The neurons were then stimulated with 200 action potentials (APs) delivered at frequencies of 10–40 Hz. The fluorescence intensity of pHluorin was monitored over time via live imaging to track SV exo- and endocytosis. For experiments comparing the kinetics of SV exo- and endocytosis, the average time course of the Syt1 response was recorded following stimulation with 200 APs at 10 Hz. The fluorescence changes were normalized to the stimulation peak, and comparisons were made between neurons stained with the Syt1 pHluorin Nb and those overexpressing Syt1 pHluorin. No differences in the kinetics were observed between the two conditions at this stimulation frequency. Fluorescence recovery to baseline was quantified at 120 s poststimulation from the fluorescence transients obtained. The average time course of the Syt1 response after stimulation with 200 APs at 40 Hz was recorded and normalized to the stimulation peak. To assess the relative amount of SV exocytosis, fluorescence changes were normalized to the total Syt1 pool, which was revealed by the application of NH_4_Cl, following stimulation with 200 APs at 10 Hz. The surface expression of Syt1 pHluorin was assessed by measuring the fraction of Syt1 pHluorin on the cell surface, with fluorescence normalized to 1 in NH_4_Cl buffer and 0 in acidic buffer. The fluorescence data are presented as the means ± standard errors of the means (SEMs). N values represent the number of individual coverslips from three separate neuronal preparations, as indicated in the plots. Statistical significance in these experiments was determined via a two-tailed Student’s t test, with significance thresholds set at ns *P* > 0.05 and ∗*P* < 0.05.

For additional experiments, live and actively recycled neurons were incubated for 15–30 min or transfected with Syt1 pHluorin. The fluorescence intensity of pHluorin was monitored over time via live imaging to track SV exo- and endocytosis. The cultures were continuously perfused with imaging buffer (mM concentrations: 136 NaCl, 2.5 KCl, 2 CaCl_2_, 1.3 MgCl_2_, 10 glucose, and 10 HEPES, pH 7.4). Imaging was performed on an inverted Zeiss Axio Observer Z1 microscope equipped with a 40x EC Plan-Neofluar oil immersion objective (NA 1.3), a Colibri 7 LED light source, and an AxioCam 506 camera controlled by ZEISS ZEN 2 software. Transfected neurons were imaged at 2 Hz via a GFP filter (excitation: 450–490 nm; beam splitter: 495 nm; emission: 500–550 nm). For experiments comparing the kinetics of SV exo- and endocytosis, the average time course of the Syt1 response was recorded following stimulation with 200 action potentials (APs) at either 10–40 Hz. The fluorescence changes were normalized to the stimulation peak, and comparisons were made between neurons stained with the Syt1 pHluorin Nb and those overexpressing Syt1 pHluorin. Fluorescence recovery to baseline was quantified at 120 s poststimulation from the fluorescence transients obtained. To assess the relative amount of SV exocytosis, fluorescence changes were normalized to the total Syt1 pool, which was revealed by the application of NH_4_Cl (50 mM), following stimulation. The surface expression of Syt1 pHluorin was assessed by measuring the fraction of Syt1 pHluorin on the cell surface, with fluorescence normalized to 1 in NH_4_Cl buffer and 0 in acidic buffer (substituting 20 mM MES for HEPES, pH 5.5). The fluorescence data were processed offline via the Time Series Analyzer plugin for ImageJ (https://imagej.nih.gov/ij/plugins/time-series.html) and are presented as the means ± standard errors of the means (SEMs). N values represent the number of individual coverslips from three separate neuronal preparations, as indicated in the plots. Statistical significance in these experiments was determined via a two-tailed Student’s t test, with significance thresholds set at ns *P* > 0.05 and ∗*P* < 0.05.

### In vivo pH calibration and resting pH estimation

The in vivo pH calibration profile and resting pH were estimated according to ^27^ with some modifications. In addition, under the assumption that H^+^ and Cl^−^ ions can move freely when the membranes are perfused with blockers and ionophores, the pH^−^ concentration inside the probe containing SV should be equal to that of the external bath solution. The current technique has been used in virtually all previous pH studies in the literature [27, 78, 79].

To calibrate the internalized pH-sensitive probes, neurons were treated with an ionophore cocktail containing the proton uncoupler FCCP, the K^+^ ionophore valinomycin, the V-ATPase inhibitor bafilomycin A1, the membrane solubilizer Triton X-100, and the K^+^/H^+^ exchanger nigericin to perfuse the membranes in high-K^+^ (122.4 mM) buffers adjusted to a defined pH. Images acquired at each pH were analyzed via bouton analysis. A pH-calibration profile was then plotted to obtain the pKa of each pH probe.

To estimate the intravesicular pH, neurons preloaded with the pHluorin/mOrange 2 probes were treated with the TEV protease for 3 min to remove all the external fluorescence signals outside the SVs, which was defined as the “resting state”. They were then very briefly incubated in ionophores containing two pH-adjusted buffers—the maximum and minimum pH buffers—on the basis of the pH calibration curve. The images were captured immediately and referred to as ‘max’ and ‘min’. Furthermore, cells in the same region of interest (ROI) were immunostained with primary labeled Abs against vGlut and vGAT to differentiate the synaptic populations into glutamatergic and GABAergic populations. Anti-vGLUT1 (guinea pig polyclonal, used at 1:500 in IF, Synaptic Systems, cat. #135304, RRID: AB_887878) and anti-VGAT (Synaptic Systems, cat. #131103, RRID: AB_887870, luminal domain) antibodies were used. The ROI was preserved by performing the experiment on top of the microscope stage and saving the X-Y position for every image that was captured.

These images were analyzed and fitted with the following equation:

$$\begin{aligned} &F \:max \\&{\mathrm{1}} + {\mathrm{1}}0pKa - pH \\&F = F + {\text{ }}0 \end{aligned}$$

The pKa was calculated from the pH calibration data. F is the fluorescence readout under ‘resting’ conditions, whose pH is unknown. F0 and Fmax are the fluorescence values at the minimum and maximum pH, respectively, whose pH is known; thus, the unknown resting pH is calculated.

### APEX2 labeling for mass spectrometry

APEX2 labeling was performed with minor modifications as previously described [28]. As a negative control, NbALFA-APEX2, which does not recognize any tag in WT neurons, was used. Biotin-phenol was prepared by dissolving 50 mg BP (Sigma SML2135-50MG) in 276 µl DMSO, followed by sonication in a water bath for 20 min to complete dissolution. This yielded a 500 mM BP stock, which was further diluted in 10 µl aliquots and stored at -80 °C until use. For H_2_O_2_ labeling, a fresh 100X stock was prepared for each use by adding 2 µl of a 30% H_2_O_2_ solution to 192 µl of Tyrode’s solution to obtain a 100 mM stock. This stock was diluted 1:100 in the labeling reaction to a final concentration of 1 mM. The quencher solution consisted of 10 mM sodium ascorbate, 5 mM Trolox, and 10 mM sodium azide in Tyrode’s solution. Sodium ascorbate was freshly dissolved in Millipore water to a concentration of 1 M, while Trolox was similarly prepared at 500 mM and sonicated. A 1 M stock solution of sodium azide was prepared, and aliquots were frozen at -20 °C. To prepare the quencher solution, 19.4 ml of Tyrode’s mixture was added to 200 µl of freshly prepared sodium ascorbate and Trolox, after which the sodium azide stock was frozen. This solution was freshly prepared because it is only effective for a short period. For these experiments, primary neuron cultures were prepared in 10 cm plates at a concentration of 5 M per plate and subjected to a BP labeling protocol. The media were removed from each plate and stored at 37 °C, leaving 1 ml of media on the cells. Three labeling cocktails were prepared with BP at a 1:500 dilution in preconditioned media in the presence of Nb-APEX2, the Nb control or without nanobody addition. These were then drop-labeled onto the neurons. After incubation for 30 min in an incubator with gentle shaking, the neurons were washed three times with their original medium and then with BP buffer (20 ml Tyrode with 20 µl BP). A fresh H_2_O_2_ pulse solution was prepared by adding 80 µl of 100X H_2_O_2_ stock solution to 8 ml of BP buffer, and 2 ml of this solution was applied to each Petri dish. After incubation for 30 s, the H_2_O_2_ solution was removed, and the reaction was quenched by adding 2 ml of the prepared quencher solution. Later, the previous quencher mixture was replaced with 1 ml for cell scraping. The cells collected in the quencher mixture were pelleted by centrifugation at 2000 rpm for 2 min, and the supernatant was discarded. The resulting cell pellets were snap-frozen and stored at -80 °C for subsequent lysis and bead enrichment procedures.

### Bead enrichment of biotinylated proteins

For cell lysis, 20 mL of RIPA lysis buffer was freshly prepared with Millipore water. The buffer contained 50 mM Tris, 150 mM NaCl, 0.1% SDS (wt/vol), 0.5% sodium deoxycholate (wt/vol), 1% Triton X-100 (vol/vol), a protease inhibitor cocktail, 1 mM PMSF, 5 mM Trolox, 10 mM sodium azide, and 10 mM sodium ascorbate. The pH was then adjusted to 7.5 with HCl, and the buffer was stored at 4 °C. Prior to lysis, the sample was precooled to 4 °C, and liquid nitrogen and ice were provided. The cells were lysed in freshly prepared RIPA lysis buffer, with ~ 300–400 µl per cell pellet. The lysates were gently pipetted and kept on ice or at 4 °C throughout the process to preserve protein integrity. After 5 min of incubation on ice, the streptavidin magnetic bead mixture was washed with 1 mL of RIPA lysis buffer, with two washes per 50 µL used per experimental set. The lysates were then cleared via centrifugation at 15,000 × g for 10 min at 4 °C. A fraction of the total homogenate (50–80 µl) was removed and snap-frozen for later MS processing and analysis. The remaining 300 µl of the sample was incubated with the washed beads for 2 h at RT with rotation. After incubation, the beads were pelleted via a magnetic rack, and the supernatant was retained and snap-frozen for future use, if needed. The beads were then thoroughly washed with 1 ml of RIPA lysis buffer at 4 °C. The beads were completely resuspended, spun on a magnetic rack and pelleted. After washing, all the wash buffer was removed before the next wash. This process was repeated twice with RIPA lysis buffer, and the samples were transferred to a new tube for each resuspension. Subsequent washes were performed with 1 ml of 1 M KCl, 1 ml of 0.1 M Na_2_CO_3_, 1 ml of 2 M urea in 10 mM Tris-HCl at pH 8.0, and two additional washes with 1 ml of RIPA lysis buffer. All wash buffers were kept on ice during the washing steps to ensure sample stability.

### EM confirmation of NbLumSyt1-APEX2 localization

Following the biotinylation step, the samples were washed thoroughly with PBS to remove any unreacted biotin-phenol and residual quenching agents. This washing step was repeated multiple times to ensure the complete removal of any nonspecifically bound substances. The samples were then incubated with a solution of streptavidin conjugated to horseradish peroxidase (HRP) at a concentration of 1 µg/mL for one hour at room temperature with gentle agitation. This incubation step allowed the streptavidin-HRP complex to bind specifically and efficiently to the biotinylated proteins, facilitating subsequent detection steps. The cells were immobilized with 2.5% glutaraldehyde in 0.1 M cacodylic buffer (pH 7.4, Electron Microscopy Sciences) for one hour at room temperature. Fixation was completed at 4 °C overnight. The cells were subsequently washed three times with ddH2O to remove the fixative, followed by incubation with DAB (1 mg/ml, Electron Microscopy Sciences) in ddH2O for 5 min on ice. Next, the supernatant was discarded, and the cells were incubated in an ice-cold solution of DAB (1 mg/ml) with H_2_O_2_ (final concentration of 5.88 mM) in ddH2O for 30 min on ice. For postfixation and staining, the cells were incubated in 1% OsO4 for approximately 2 min on ice. The cells were subsequently washed several times with ddH2O and dehydrated with a graded ethanol series (30, 50, 70, 100%) with two final dehydration steps in propylene oxide for 5 min each. Finally, the cells were infiltrated with EMBed-812 resin (Science Services) and cured at 60 °C for 48 h. TEM micrographs of thin Sects. (60–70-nm-thick) were recorded on a Phillips CM120 transmission electron microscope equipped with a 2k × 2k slow-scan CCD camera (TVIPS) and operated at 120 kV.

### LC‒MS/MS analysis

MS analysis of the digested proteins was performed in three biological replicates, each analyzed in technical duplicates, via a hybrid quadrupole-ion trap-orbitrap mass spectrometer (Orbitrap Fusion, Thermo Fisher Scientific, San Jose, USA). Data acquisition was performed via a data-dependent acquisition method. Peptides were first concentrated on a C18 PepMap100 trapping column (0.3 mm × 5 mm, 5 μm, Thermo Fisher Scientific, Waltham, USA) and subsequently separated on an in-house packed C18 analytical column (75 μm × 300 mm, Reprosil-Pur 120 C18-AQ, 1.9 μm, Dr. Maisch GmbH, Ammerbuch, Germany). Liquid chromatography was performed on an UltiMate 3000 UHPLC nanosystem (Thermo Fisher Scientific, Waltham, USA) with columns preequilibrated in a mixture of 95% buffer A (0.1% v/v formic acid in water) and 5% buffer B (80% v/v acetonitrile with 0.1% v/v formic acid in water). Peptides were eluted over a 118-minute gradient from 5 to 50% buffer B, followed by washing with 90% buffer B for 6 min and re-equilibration with 5% buffer B for another 6 min. The mass spectrometer was operated in DDA mode with MS1 scans covering a 350–1650 m/z range at a resolution of 120,000 at 200 m/z, with a 300% automated gain control (AGC) target and a maximum injection time of 50 ms. Peptide ions with charge states of 2–7 were selected for MS/MS analysis via a 1.6 m/z isolation window. Fragmentation was induced by HCD at 28% of the normalized collision energy (NCE); fragments were detected in the Orbitrap at a resolution of 15,000 at 200 m/z, with an AGC of 1000% normalized and a maximum injection time of 54 ms. The duty cycle was maintained at 2.5 s with dynamic exclusion of 30 s. The analysis of the raw data was subsequently performed in MaxQuant via the *Rattus norvegicus* proteome (UP000234681), supplemented by sequences of the APEX2 nanobodies as positive controls, for validation via IP. For statistical analysis of the data, the label-free quantification (LFQ) intensities of the proteins found to be significantly enriched vs. both the input IP and the negative control IP (a NbALFA-APEX2 IP or a no-Nanobody IP) were selected. For initial analyses, we relied on unbiased gene ontology (GO) CC analysis [80]. Since several potential interactors were found, we decided to concentrate on the targets that were strongly enriched both vs. the controls (> 1500 enrichment vs. the controls) and enriched even compared with the initial input (> 1.5 enrichment vs. the input). Only a few proteins passed this stringent cutoff, including Cntfr, immunoglobulin superfamily member 8 (Igsf8), integral membrane protein 2B (Itm2b) and heat shock 70 kDa protein 13 (Hspa13).

### Precomplex formation of nbSyt1 nanobodies for uPAINT imaging

The NbLumSyt1-pH/Nb-Atto647N and NbLumSyt1-HALO/JF549 complexes were freshly prepared before each experiment. To prepare the NbLumSyt1-pH/nb-At647N complex, a 110-fold molar excess of NbLumSyt1-pH was mixed with anti-GFP NbLumSyt1-At647N (camelid sdAB from Synaptic Systems, N0301-At647N-S) at 4 °C for 16 h, covered from light, with gentle rotation (20 rpm). The NbLumSyt1-pH/nb-At647N complex was then centrifuged at 18,000 × g for 10 min at room temperature to remove precipitates, subsequently diluted (1:200) in high-K^+^ buffer, mixed at room temperature for 5 min with gentle rotation (20 rpm), and used for imaging on the same day. To prepare the NbLumSyt1-HALO/JF549 complex, a 110-fold molar excess of NbLumSyt1-HALO was mixed with JaneliaFluor 549 HaloTag ligand for super-resolution microscopy (Promega, GA1110) at room temperature (i.e., 22–25 °C) for 30 min, covered from light, with gentle rotation (20 rpm). The NbLumSyt1-HALO/JF549 complex was then centrifuged at 18,000 × g for 10 min at RT to remove precipitates, subsequently diluted (1:200) in high-K^+^ buffer, mixed at RT for 5 min with gentle rotation (20 rpm), and used for imaging on the same day. These approaches ensure a 1 nM effective concentration of the fluorescently labeled nanobody (NbLumSyt1-pH/Nb-At647N or NbLumSyt1-HALO/JF549) on the imaging plate, allowing partial labeling of the Syt1 nanobodies without oversaturating the fluorescent signal and enabling single-molecule detection.

### Single-molecule imaging by uPAINT

Single-molecule uPAINT experiments to detect and track endogenous and overexpressed Syt1 on the plasma membrane were performed as described previously [33, 81]. Neurons at 21–22 days in vitro (DIV) were washed and stimulated in high-K+ buffer supplemented with 1 nM complexes, engineered to bind endogenous Syt1, and imaged in HILO for 320 s at 50 Hz. For comparison, hippocampal neurons were transfected with Syt1-pHluorin (Syt1-pH), and uPAINT imaging was performed via high-K+ buffer supplemented with 1 nM anti-GFP nb-At647N, which specifically binds to the pHluorin moiety. Time-lapse imaging was carried out at 50 Hz and 20 ms exposure at 37 °C on an Roper microscope (Roper Scientific) equipped with an iLas2 double-laser illuminator, a CFI Apo TIRF 100 × 1.49 NA objective (Nikon) and an Evolve512 delta EMCCD camera (Photometrics, model no. Evolve 512 Delta). A quadruple beam splitter (LF 405/488/561/635-A-000-ZHE; Semrock) and a QUAD band emitter (FF01-446/510/581/703 − 25; Semrock) were used, and image acquisition was performed via MetaMorph Microscopy Automation and Image Analysis Software (v7.7.8, Molecular Devices). The single-molecule localization and dynamics data were extracted from 16,000 frames acquired in HILO as previously described [82]. Endogenous Syt1-bound NbSyt1-pH/Nb-At647N and nbSyt1-HALO/JF549 complexes and Syt1-pH-bound nb-At647N were detected and tracked via a combination of wavelet segmentation [83] and optimization of multiframe object correspondence via simulated annealing [84]. Single-molecule localization and tracking were performed with PALMTracer [85] software within MetaMorph Software. We applied a cross-correlation-based drift correction to the data via the SharpViSu tool [86], and tracks shorter than seven frames were excluded from the analysis to minimize nonspecific background. The color coding for the super-resolved images was performed via Fiji/ImageJ [65] (2.0.0-rc-68/1.52 h; National Institutes of Health). Diffusion coefficients were calculated for each trajectory and presented in a color-coded pixel at the site of localization. In the average intensity maps, each pixel indicates the localization of an individual detected molecule. The area with the highest density is represented in white. The color coding of the trajectory maps is arbitrary and corresponds to the colors shown in the graphs. The statistics for these experiments considered normality and lognormality, as well as AUC and frequency distribution quantifications. These tests were carried out in Prism 8 for macOS software (version 8.3.0). Statistical analysis of normally distributed data was performed via one-way ANOVA for multiple comparisons, and for nonnormally distributed data, one-way ANOVA with the Kruskal‒Wallis test was used for multiple comparisons. Error bars represent ± SEM for independent experiments, and individual dots in the scatter plot graphs represent individual neurons. *N* = 22 neuronal cultures from 3 independent experiments in each condition. For DBSCAN, 10 representative datasets from nbSyt1-HALO/JF549 experiments were analyzed. Nonsignificant differences are indicated with n.s., and asterisks indicate the following p values: *p < 0.05, ** *p* < 0.01, and **** *p* < 0.0001. *N* = 22 neuronal cultures from 3 independent experiments in each condition.

### Nanocluster analysis

Nanoclustering analysis of Syt1 uPAINT data was performed via the Python-based nanoscale spatiotemporal indexing clustering (NASTIC) workflow [36]. NASTIC determines clustering on the basis of overlapping spatiotemporal bounding boxes of each tracked single molecule in space and time. Briefly, trajectories within a region of interest (ROI) were screened to remove those with fewer than 8 sequential detections. Spatial centroids were determined for each trajectory by averaging the x and y coordinates of all the detections. A convex hull of all the detections associated with the trajectory was calculated, and the radius R was derived from its area. An idealized 3D spatiotemporal bounding box with [x, y,z] dimensions x = 2R*r*, y = 2R*r* and z = *t* was created around each centroid, where *r* = 1.2 and *t* = 20 s. These *r* and *t* values have been previously empirically determined to best yield clustering metrics reflecting the ground truth of a range of synthetic trajectory datasets, as well as experimentally derived single-molecule imaging data. The bounding boxes were indexed into a 3D R-tree spatial database, which was then queried to return overlapping bounding boxes. Overlapping bounding boxes were then used to assign their parent trajectories into clusters. For each NASTIC cluster, a convex hull of all the detections comprising the clustered trajectories was used to determine the cluster area, radius and subsequent metrics. Clusters with radii > 150 nm were screened from the analyses.

### MoNaLISA imaging scheme and acquisition

The MoNALISA setup used in this study was custom-built as previously reported [39]. Briefly, images were recorded with a multifoci pattern with a periodicity of 625 nm coupled with an OFF pattern of 312.5 nm. The ON switch was performed with 405 nm light at 650 W/cm^2^ for 0.5 ms, the OFF was confined with 4 ms of 488 nm light at 650 W/cm^2,^ and finally, the “read out” was performed with 240 kW/cm^2^ of 488 nm light per 1 ms. The step size was 35 nm. In the case of two-color recording, the redshifted channel was imaged in a sequential manner on a second camera with a 620/70 nm interval. A 350 W/cm^2^ of 590 nm light per 2 ms was used for the recording. We applied bleaching correction between frames to compensate for switching fatigue. The images presented were deconvolved with a narrow Gaussian of 50 nm FWHM combined with a wider Gaussian of 175 nm FWHM accounting for 10% of the PSF amplitude; such a geometry considers the properties of rsFPs, where a background signal due to a nonswitchable fraction of the molecules is expected. The final image is the result of 5 iterations of the Richardson-Lucy algorithm.

### MoNaLISA image analysis

To detect the clusters of Syn1 from the MoNALISA images, the following pipeline was used. First, to identify the clusters, the deconvolved (5 iterations) and bleached corrected images were filtered via a combination of a Laplacian filter (3 pixels) to increase their contrast and a Gaussian filter (1 pixel) to reduce the background. After a global threshold (common to the whole dataset), the clusters can be defined, and parameters such as area, intensity and ellipticity can be extracted. Clusters closer to 70 nm were merged. To further understand the inner composition of the clusters, we identified the maxima for each cluster (given a tolerance parameter of 5). In the case of two-color recording, the condition of the inhibitory cluster does not depend on the overlap or proximity (less than 70 nm) of the clusters in the two channels.

### hiPSC differentiation into hypothalamic neurons

The control L2135 iPSC line [87, 88] was differentiated into hypothalamic neurons according to a protocol described previously [89] with minor modifications. Briefly, iPSCs were maintained in Matrigel-coated plates with mTeSR1 (Stem Cell Technologies), and the medium was changed every two days. For hypothalamic neuron differentiation, the cells were plated on Matrigel-coated plates in N2B27 medium (Neurobasal A: DMEM/F12 (1:1) (Life Technologies), 1x B27 without Vit. A (Life Technologies), 1x N2 (Life Technologies), 1x PenStrep, 1x GlutaMAX (Life Technologies), 0,075% sodium bicarbonate (Life Technologies), 0.5x MEM-NEAA (Life Technologies), 200 nM ascorbic acid (Sigma)) supplemented with SB431542 (Tocris, 10 µM), XAV939 (VWR, 2 µM) and LDN-193,189 (Sigma, 100 nM). The medium was changed every 2 days, and the supplement concentration was gradually reduced. To ventralize the progenitors, 1 µM SAG (Merck) and 1 µM purmorphamine (Sigma) were added between day 2 and day 8. DAPT (Tocris, 5 µM) was added from day 8 to day 14. After 14 days, the cells were trypsinized and replated with N2B27 medium on poly-D-lysine/laminin-coated 16–18 mm coverslips for terminal differentiation in the presence of BDNF (R&D Systems, 10 ng/ml).

### Syt1 nanobody uptake by human stem cell-derived neurons

Eighty-day-old human hypothalamic neurons were used for the uptake experiments. All nanobody incubations were performed in BrainPhys medium (StemCell Technologies) to increase synaptic activity, and the diluted nanobodies were centrifuged in a tabletop centrifuge at maximum speed for 10 min at room temperature. The surface Syt1 pool was blocked by incubating the neurons with 4.85 µM unlabeled Syt1 nanobody for 5 min at 37 °C in a humid chamber. After being washed 3 times with PBS, the neurons were incubated with 5.5 µM pHluorin-labeled Syt1 nanobody in the presence of either 30 mM KCl or 1 µM TTX (Alomone Lab) to stimulate the neurons or to silence them, respectively, for a total of 20 min at 37 °C in a humid chamber. The cells were extensively washed with PBS, fixed in 4% PFA for 15 min, permeabilized, blocked with 0.01% saponin/10% normal goat/PBS, and stained with primary antibodies in the same blocking solution in a humid chamber. The primary antibodies used were anti-GFP (Aves labs GFP-1020, 1:1000), anti-Syt1 (Synaptic Systems 105–011; 1:100) and anti-TUBB3 (Biolegend Poly18020; 1:1000). All secondary antibodies were Alexa Fluor-conjugated (Thermo Fisher Scientific; 1:500). Nuclei were counterstained with DAPI (Sigma‒Aldrich). Images were acquired with a Nikon A1R confocal microscope through a 60X NA1.2 water immersion lens, and the signals were quantified with Fiji via the Coloc2 plugin (https://imagej.net/plugins/coloc-2).

### iNeuron culture

iNeurons were obtained from the iPSC line BIHi005-A (generated at the Max Delbrück Center) as described previously [41]. After 20 days, the neurons were fed weekly by replacing 1/10 the volume of the old media with fresh growth media containing 2 µM Ara-C. For activity measurements, neurons (6–8 weeks in culture) were stimulated with 200 action potentials at 40 Hz for 5 s via a field stimulation chamber RC-47FSLP (Warner Instruments) and imaged at physiological temperature (37 °C) in imaging buffer [90] containing 1.3 mM Ca^2+^ with an epifluorescence microscope Nikon Eclipse Ti equipped with a 40X oil objective. Images were acquired every 2 s. Analysis was performed with SynActJ [91].

## Supplementary Material

Below is the link to the electronic supplementary material.

Supplementary Material 1

Supplementary Material 2

## Data Availability

Data is provided within the manuscript or supplementary information files.
