# Supplementary material for: A versatile nanobody platform for live and super-resolution imaging of synaptic vesicle dynamics and plasticity in rodent and human neurons
Source: J Nanobiotechnology. 2026 May 14;24:443. doi: 10.1186/s12951-026-04489-w (PMC13185310; doi:10.1186/s12951-026-04489-w)
Supplement: Supplementary file 2 — Supplementary Material 2 [file 12951_2026_4489_MOESM2_ESM.docx]

**Supplementary Figures**


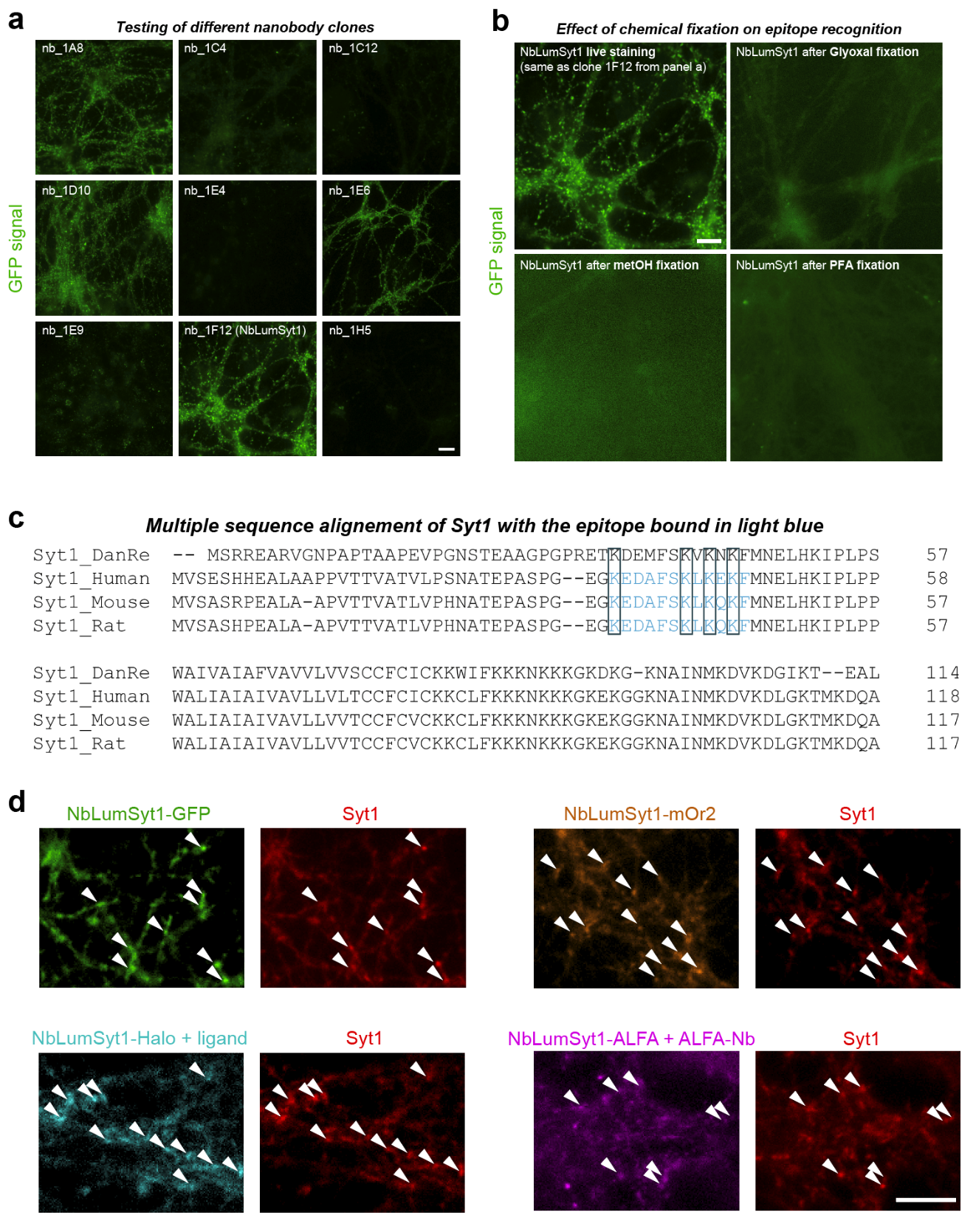


***Supplementary Fig. 1: Nanobody clone selection and initial characterization. a****) Testing the nanobody clones (fused to GFP) that were found to bind the Syt1 epitope via ELISA via live immunofluorescence in primary hippocampal neurons. Among the tested candidates, 1A8, 1D10, 1E6 and 1F12 showed the most promising binding. After analysis of the protein sequence of the nanobodies, these four clones appeared highly related (not shown); hence, clone 1F12, which seemed to be the strongest binder, was selected for further characterization.* ***b****) Upon chemical fixation, the ability to recognize the epitope is lost.* ***c****) Protein sequence conservation analysis of the epitope bound by the nanobody reveals that this region is conserved and presents several lysins (K), which, upon fixation due to the reactivity of their primary amine group, can form methylene bridges with other surrounding molecules and lose their initial immunogenic properties.* ***d****) Colocalization of different nanobody fusion constructs upon live labelling of primary neurons which were fixed and counterstained with the anti-Syt1 cytoplasmatic domain (SySy 105 011). Scale bars: 10 µm.*

***
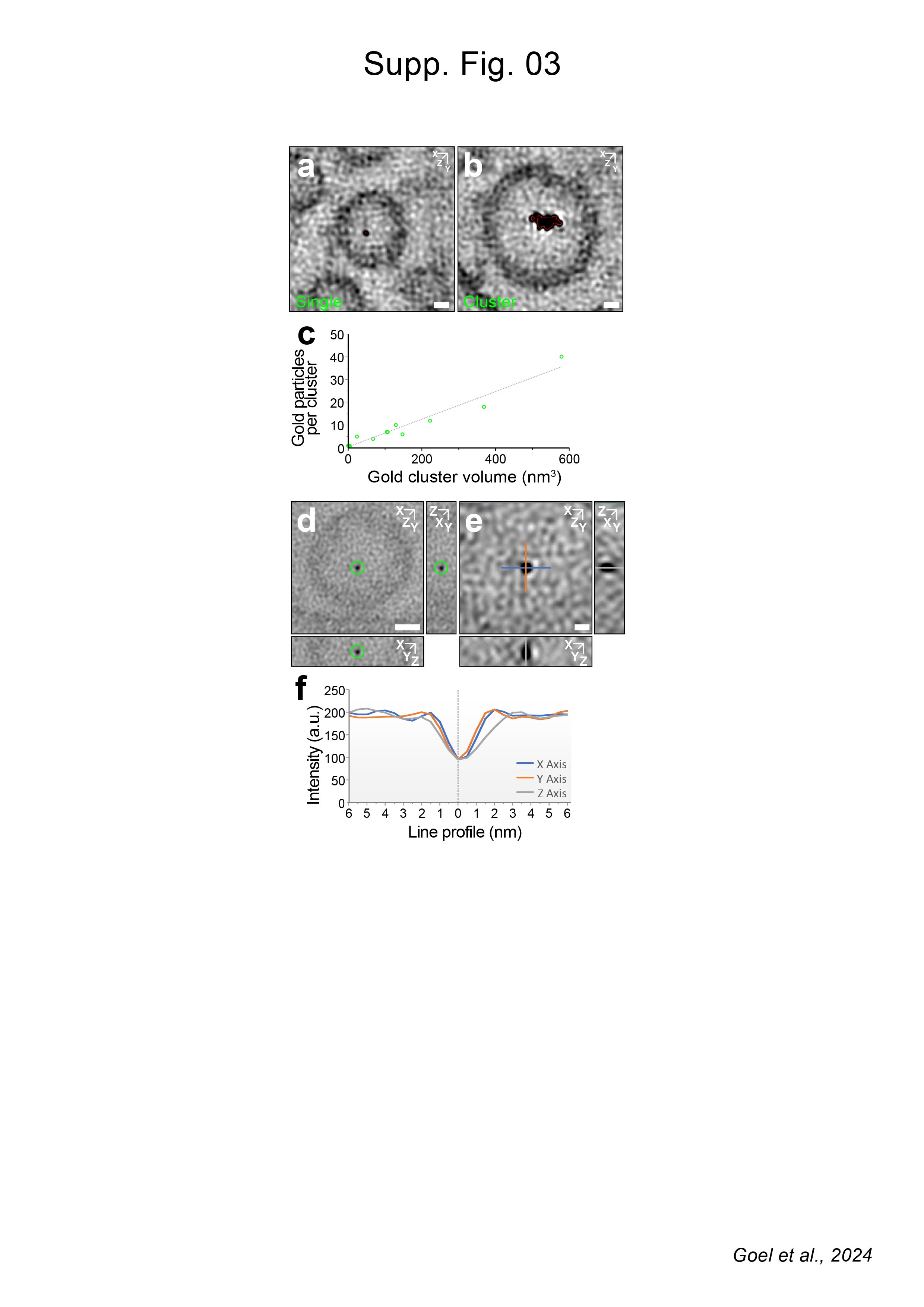
***

***Supplementary Fig. 2: Additional measures related to Figure 2.*** ***a, b****) Electron tomographic subvolumes acquired at 36000x magnification (voxel size x,y,z = 1.2 nm; z-projection = 5 slices) illustrating gold-labeled clear-core synaptic vesicles (CCSV^G^) from the NbLumSyt1-ALFA-tag:NbALFA-Gold complex (NbLumSyt1)-treated synapses. A single luminal gold particle (a) and a cluster (b) are shown (red outline indicates automatic segmentation of gold used to estimate luminal gold-labeled volumes).* ***c****) Plot demonstrating the linear relationship between the number of manually quantified luminal gold particles and corresponding unbiased volume estimates in individual vesicles from a tomogram acquired from an NbLumSyt1-ALFA-tag:NbALFA-Gold complex-treated synapse.* ***d-f****) Electron tomographic subvolume acquired at 57000x magnification (voxel size x,y,z = 0.5 nm; z-projection = 6 slices) from an NbLumSyt1-ALFA-tag:NbALFA-Gold complex-treated synapse in which a single luminal gold particle is clearly resolved (****d****). An enlargement (****e****) indicates orthogonally positioned line profiles centered on the gold particle (blue, x-axis; orange, y-axis; gray, z-axis). The corresponding intensity profiles (****f****) are consistent with the detection of an individual 3 nm gold particle. Scale bars: 10 nm in* ***a****,* ***b****, and* ***d****; 3 nm in* ***e****.*

***
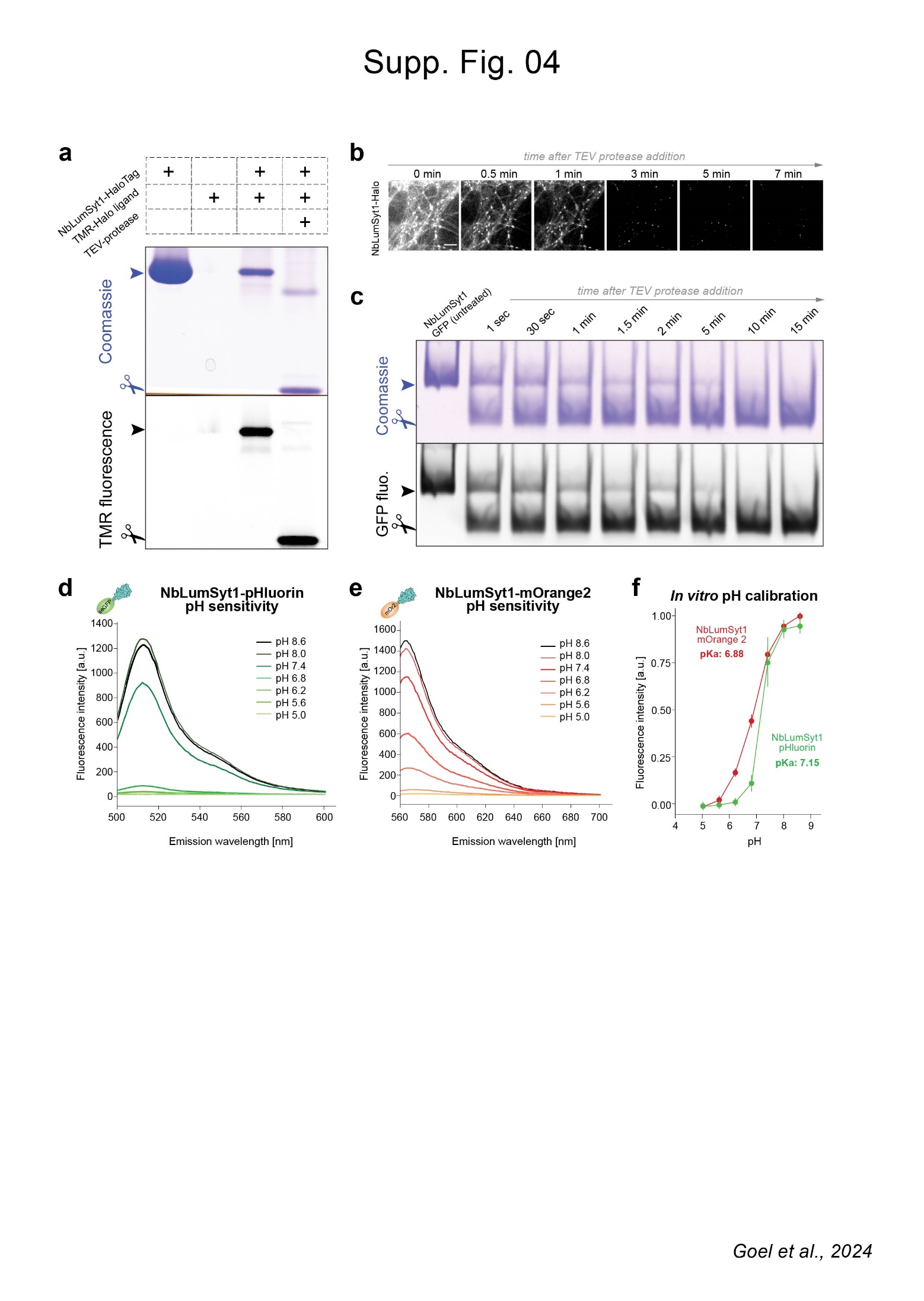
***

***Supplementary Fig. 3: TEV protease-cleavable versions of NbLumSyt1 for studying the readily retrievable pool of synaptic vesicles. a****) SDS‒PAGE analysis of NbLumSyt1-TEV-Halo, either alone or in complex with the TMR-labeled Halo ligand, before and after TEV protease treatment. Upon TEV cleavage, the NbLumSyt1-TEV-Halo/TMR ligand complex reveals two bands, with the lower band containing the Halo-Tag/Halo ligand complex and being fluorescent (lower image) and the upper band containing the separated nanobody.* ***b****) In contrast to live labeling of primary neurons with NbLumSyt1-TEV-mOrange (surface only) and TEV-protease treatment, the mOrange signal is efficiently removed from cultured neurons, indicating that the fluorescent signal is derived from surface-exposed pools since the intravesicular pools are quenched due to their low pH.* ***c****) An experiment similar to that in a) performed in isolated NbLumSyt1-TEV-mOrange reveals that the time course of cleavage resembles that of labeled neurons.* ***d-f****) Estimation of the resting pH using NbLumSyt1-pHluorin (****d****) or NbLumSyt1-mOrange2 (****e****) in isolated molecules (in vitro). For the same measurement in primary neurons, refer to the main figure. To estimate the pKa values, calibration curves were created by titrating the pH response of the probes. In accordance with previous reports ^27^, the pKa of mOrange is lower than that of pHluorin, extending the measurement range toward lower pH values.*

***
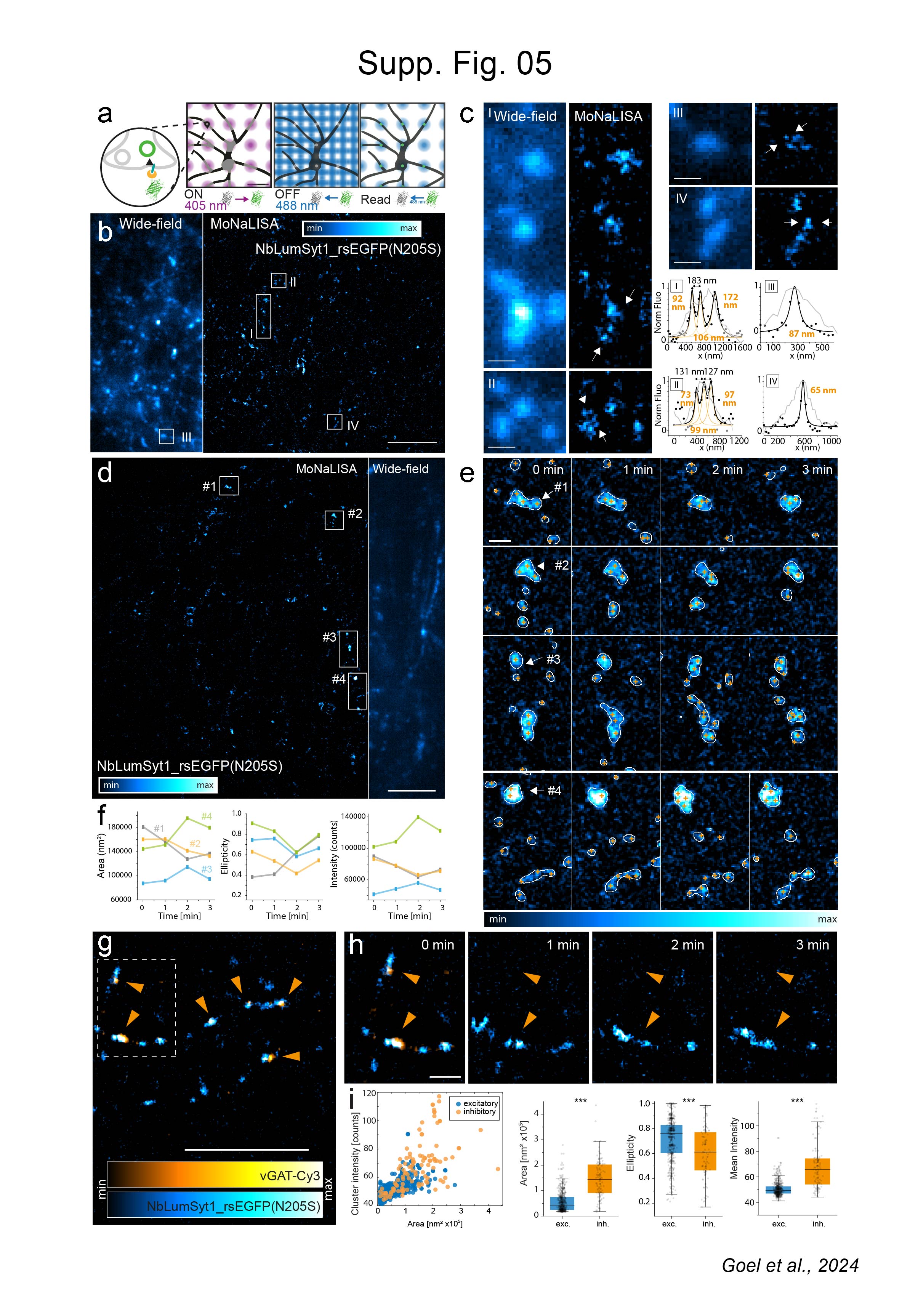
***

***Supplementary Fig. 4: MoNaLISA diffraction unlimited imaging of NbLumSyt1-rsEGFP(N205S). a****)* *Schematic illustration of the MoNaLISA imaging scheme. The illumination is parallelized over an extended field of*

*(continuing from the previous page)*

*view using multiple foci to switch in the ON state, the rsFPs (reversibly switchable fluorescent proteins) are located in an array of focal spots and sinusoidal light patterns to switch the rsFPs located in the periphery of each of the foci in the OFF state. The fine regions containing the remaining rsFPs in the ON state are then read out with an additional array of blue foci.* ***b, c****) Comparison between a wide-field image and a MoNaLISA image of live hippocampal neurons labeled with NbLumSyt1-rsEGFP (N205S). The ROIs in c show different magnifications around different regions of the field of view, reporting the resolution achieved in the visualization of the cluster through line profiles traced across the white arrows. Clusters of sizes down to 65 nm in width can be isolated with separation down to 130 nm.* ***d, e****) Time-lapse imaging of NbLumSyt1-rsEGFP(N205S) at intervals of 1 min between images.* ***f****) Syt1 clusters can increase (#4) or decrease (#1) in area, number and overall shape of the active zone within minutes. The graphs follow area, ellipticity and intensity for 4 clusters out of the 73 identified clusters in the same field of view.* ***g-i****) Dual color imaging and quantification of NbLumSyt1-rsEGFP (N205S) in the cyan channel and an inhibitory synapse marker in the orange channel (vGAT-Cy3).* ***i****) Inhibitory synapses (vGAT-positive) significantly increased in area and mean intensity. The shape of the active zone of the inhibitory synapses was also mostly elliptical and packed, with an increased number of small clusters. Ellipticity is defined as the ratio between the minor axis and the major axis of each cluster (ranging from 0-1). The graphs are the results of 648 clusters from 6 experiments, with a mean of approximately 100 clusters identified per field of view. Unpaired Student’s t test. *** = p ≤ 0.001. Scale bars: 650 nm in* ***a****; 5 µm in* ***b****,* ***d****, and* ***g****; 500 nm in* ***c*** *and* ***e****; 1 µm in* ***h****.*
